# Author Correction: Crizotinib-induced immunogenic cell death in non-small cell lung cancer

**DOI:** 10.1038/s41467-019-09838-y

**Published:** 2019-04-17

**Authors:** Peng Liu, Liwei Zhao, Jonathan Pol, Sarah Levesque, Adriana Petrazzuolo, Christina Pfirschke, Camilla Engblom, Steffen Rickelt, Takahiro Yamazaki, Kristina Iribarren, Laura Senovilla, Lucillia Bezu, Erika Vacchelli, Valentina Sica, Andréa Melis, Tiffany Martin, Lin Xia, Heng Yang, Qingqing Li, Jinfeng Chen, Sylvère Durand, Fanny Aprahamian, Deborah Lefevre, Sophie Broutin, Angelo Paci, Amaury Bongers, Veronique Minard-Colin, Eric Tartour, Laurence Zitvogel, Lionel Apetoh, Yuting Ma, Mikael J. Pittet, Oliver Kepp, Guido Kroemer

**Affiliations:** 1 Faculty of Medicine, University of Paris Sud, Kremlin-Bicêtre, 94270 France; 2 Cell Biology and Metabolomics Platforms, Gustave Roussy Cancer Campus, Villejuif, 94805 France; 3 Equipe 11 labellisée Ligue Nationale contre le Cancer, Centre de Recherche des Cordeliers, Paris, 75006 France; 4 Institut National de la Santé et de la Recherche Médicale, UMR1138, Equipe labellisée Ligue Nationale Contre le Cancer, Paris, 75006 France; 5 Université Paris Descartes, Sorbonne Paris Cité, Paris, 75006 France; 6 Université Pierre et Marie Curie, Paris, 75006 France; 7 Center for Systems Biology, Massachusetts General Hospital Research Institute and Harvard Medical School, Boston, 02139 MA USA; 8 Graduate Program in Immunology, Harvard Medical School, Boston, 02238 MA USA; 9 Koch Institute for Integrative Cancer Research, Massachusetts Institute of Technology, Cambridge, 02139 MA USA; 10 Department of Radiation Oncology, Weill Cornell Medical College, New York, 14853 NY USA; 11 Department of Anaesthesiology, Hôpital Européen Georges Pompidou, Paris, 75015 France; 12 Centre de Recherche INSERM LNC-, UMR1231 Dijon, France; 13 Department of Medical Oncology, Centre Georges-François Leclerc, Dijon, 21000 France; 14 Center for Systems Medicine, Institute of Basic Medical Sciences, Chinese Academy of Medical Sciences and Peking Union Medical College, Beijing, 100730 China; 15 Suzhou Institute of Systems Medicine, Suzhou, 215123 Jiangsu China; 16 Department of Pharmacology, Institut Gustave Roussy, Villejuif, 94805 France; 17 School of Pharmacy, Université Paris Sud, Châtenay-Malabry, 92 296 France; 18 Institut de Cancérologie, Gustave Roussy Cancer Campus (GRCC), Villejuif, 94805 France; 19 INSERM U970, Université Paris Descartes Sorbonne Paris-Cité, Paris, 75006 France; 20 Department of Immunology, Hôpital Européen Georges Pompidou, Paris, 75015 France; 21 INSERM U1015, Villejuif, 94805 France; 22 Center of Clinical Investigations CIC1428, Villejuif, 94805 France; 23 Department of Radiology, Massachusetts General Hospital, Boston, 02114 MA USA; 24 Pôle de Biologie, Hôpital Européen Georges Pompidou, AP-HP, Paris, 75015 France; 25 Department of Women’s and Children’s Health, Karolinska University Hospital, Stockholm, 141 86 Sweden

**Keywords:** Cancer immunotherapy, Targeted therapies, Autophagy, Cell death, Cell death and immune response

Correction to: *Nature Communications* 10.1038/s41467-019-09415-3, published online 2 April 2019

The original version of this Article contained an error in the spelling of the author Lin Xia, which was incorrectly given as Xia Lin. This has now been corrected in both the PDF and HTML versions of the Article.

