# Supplementary material for: Crizotinib-induced immunogenic cell death in non-small cell lung cancer
Source: Nat Commun. 2019 Apr 2;10:1486. doi: 10.1038/s41467-019-09415-3 (PMC6445096; doi:10.1038/s41467-019-09415-3)
Supplement: Supplementary file 1 — Supplementary Information [file 41467_2019_9415_MOESM1_ESM.pdf]

Crizotinib-Induced Immunogenic Cell Death in Non-Small Cell Lung Cancer

Liu et al.

Supplementary materials: 19 figures; 1 table.

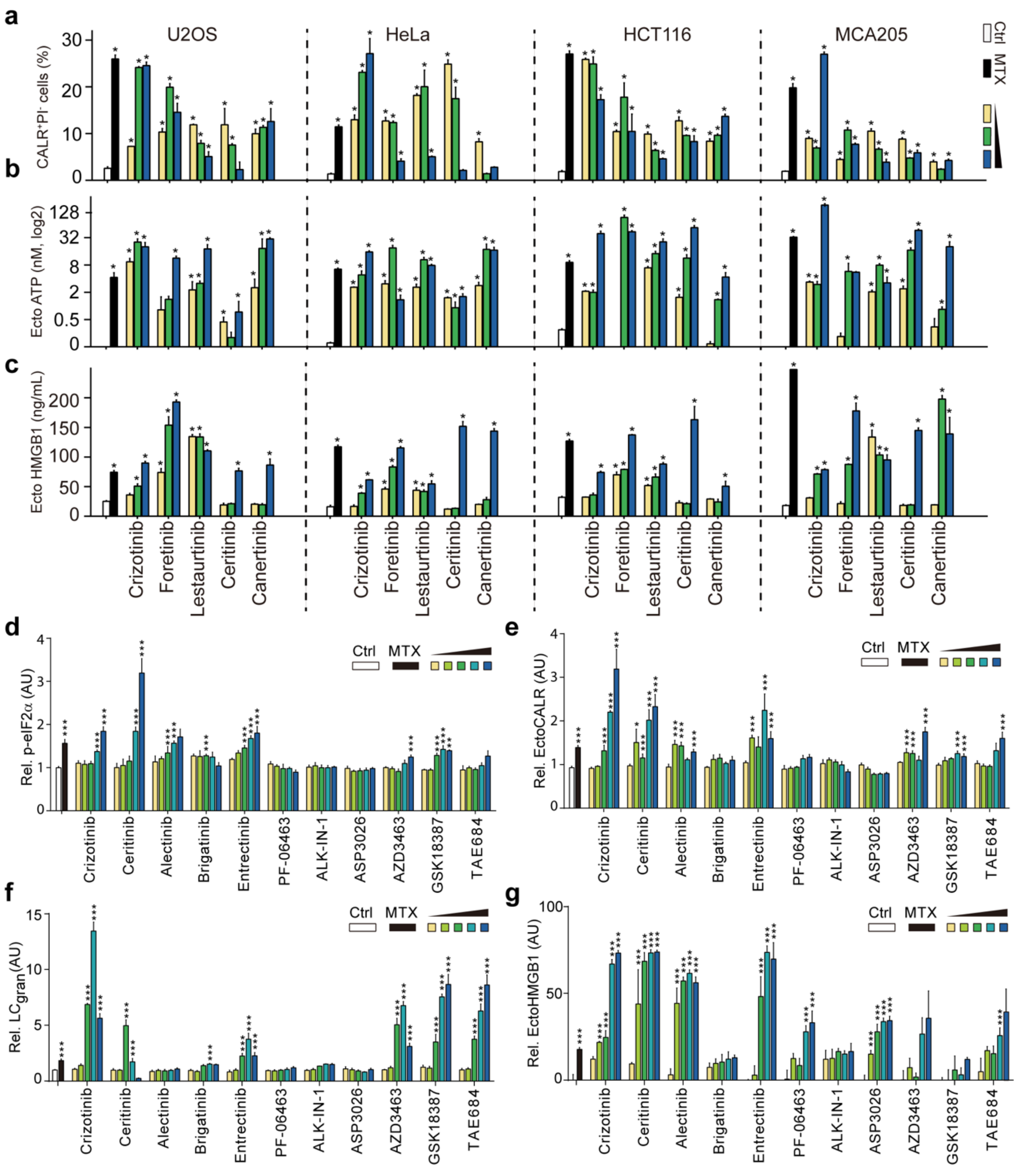

Supplementary Figure 1. Validation of ICD effects by top TKIs candidates in different cell lines

(**a-c**) Human osteosarcoma U2OS cells, cervical adenocarcinoma HeLa cells, colon adenocarcinoma HCT-116 cells and murine fibrosarcoma MCA205 cells were treated with mitoxantrone (MTX, 2 μM), or increasing concentrations (1, 5, 10 μM) of tyrosine kinase inhibitors for 8 h before determination of calreticulin (CALR) exposure, and 24 h before detection of ATP secretion and HMGB1 release (mean ± s.e.m., n = 3; *p < 0.001 compared to Ctrl, t-test). (**d-f**) U2OS cells or its derivatives co-expressing CALR-RFP and HMGB1-GFP or GFP-LC3 were treated with increasing concentrations (0.5, 1, 2.5, 5, 10 μM) of the indicated ALK inhibitors before fixation and immunostaining if needed. Fluorescence microphotographs were quantified for assessing eIF2α phosphorylation (**d**), CALR exposure (**e**), autophagy induction (f) and HMGB1 release (g). (mean ± s.e.m., n = 4; ***p < 0.001 compared to untreated control (Ctrl), t-test)

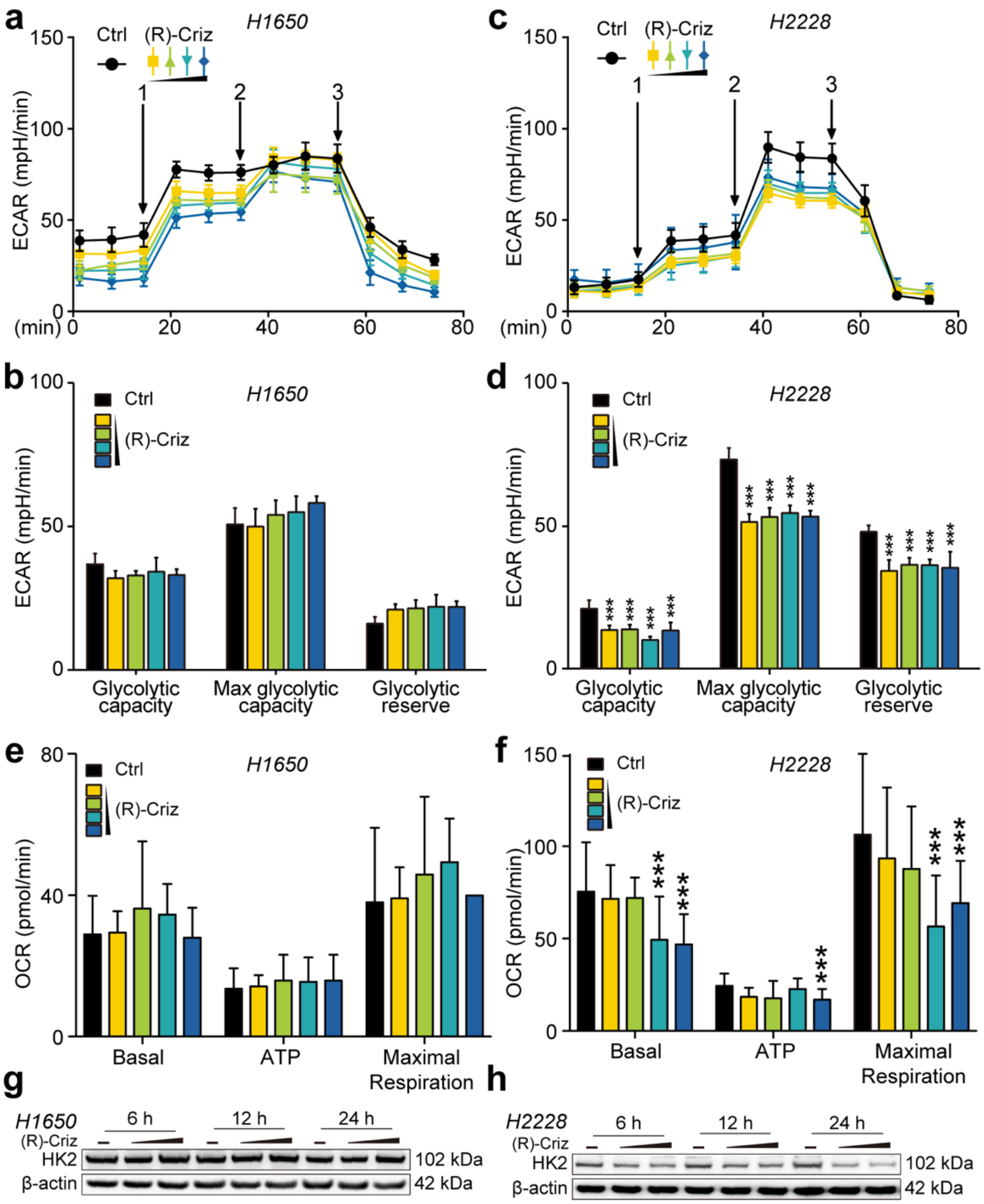

Supplementary Figure 2. Changes in glycolytic flux and mitochondrial respiration in responses to (R)-crizotinib treatment. H1650 or H2228 cells were seeded in Seahorse XF96 microplates, allowed to adapt for 24 h and were then placed in medium that contains 0.2 % DMSO or different concentrations (1, 2.5, 5, 10 μM) of (R)-crizotinib (Criz) for 6 h before metabolic assessment by means of the Seahorse analyzer. (**a**,**c**) Arrows indicate glucose (1), oligomycin (2), and 2-deoxyglucose (3) injections. Extracellular acidification rate (ECAR, **b**,**d**) and oxygen consumption rate (OCR, **e**,**f**) are reported as mean ± s.e.m. ($n = 6$; ***$p < 0.001$ compared to untreated control (Ctrl), t-test). (**g**,**h**) H1650 or H2228 cells were treated with (R)-Criz for indicated times to assess its effect on hexokinase II (HK2) expression; β-actin was used as a loading control.

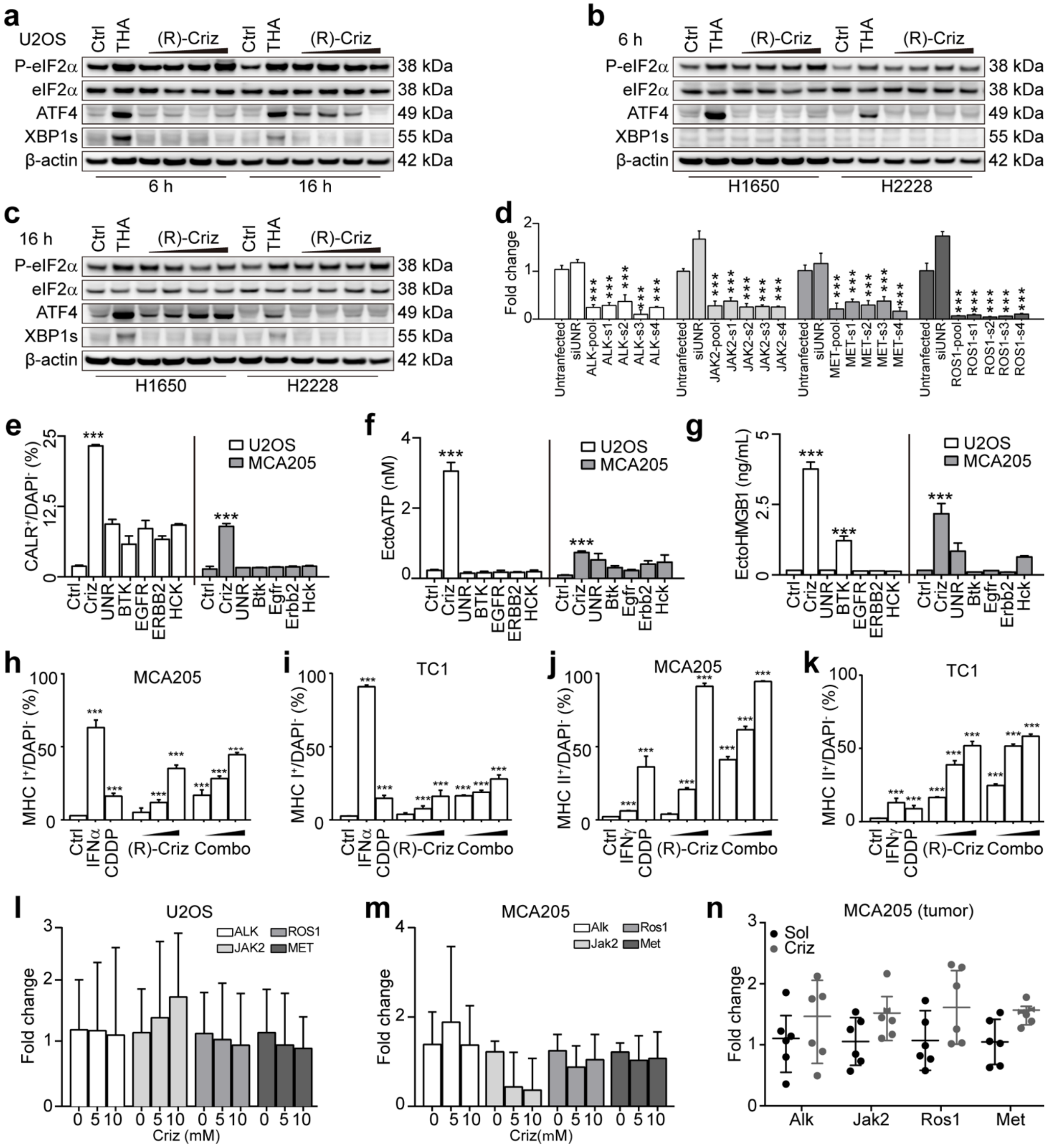

Supplementary Figure 3. Alternative mechanisms of (R)-crizotinib induced ICD. (**a-c**) (R)-crizotinib induced off-target ER stress. U2OS, H1650 and H2228 cells were treated with thapsigargin (THA, 2 μM) or different concentrations (1, 2.5, 5, 10 μM) of (R)-crizotinib (Criz) for 6 or 16 h before cells were lysed for western blotting. (**d**) Validation of siRNA-mediated gene knockdown by real-time qPCR in U2OS cells. (**e-g**) SiRNA knock-down of different kinases for ICD assessment. Indicated siRNAs (a pool of four siRNA duplexes) were reversely transfected into U2OS or MCA205 cells and ICD hallmarks (CALR exposure, ATP and HMGB1 release) were assessed. Treatment with (R)-Criz for 24 h was used as positive control. Data are shown as mean ± s.e.m. (n = 3; ***p < 0.001, compared to untreated control (Ctrl), t-test). (**h-k**) MCA205 and TC1 cells were treated by (R)-Criz (0.5, 1, 2.5 μM) alone or plus CDDP (Combo) for 24 h before surface staining with MHC class I/II antibodies.

Recombinant murine IFNα and γ were used as positive controls for MHC class I and II stimulation respectively. Data are shown as mean ± s.e.m. (n = 4; ***p < 0.001, compared to Ctrl, t-test). (**l-n**) Quantitative PCR analysis of mRNA levels *in vitro* (**l,m,** 24 h treatment) and *in vivo* (**n**, 48 h post *i.p.* injection, n = 6 animals per group).

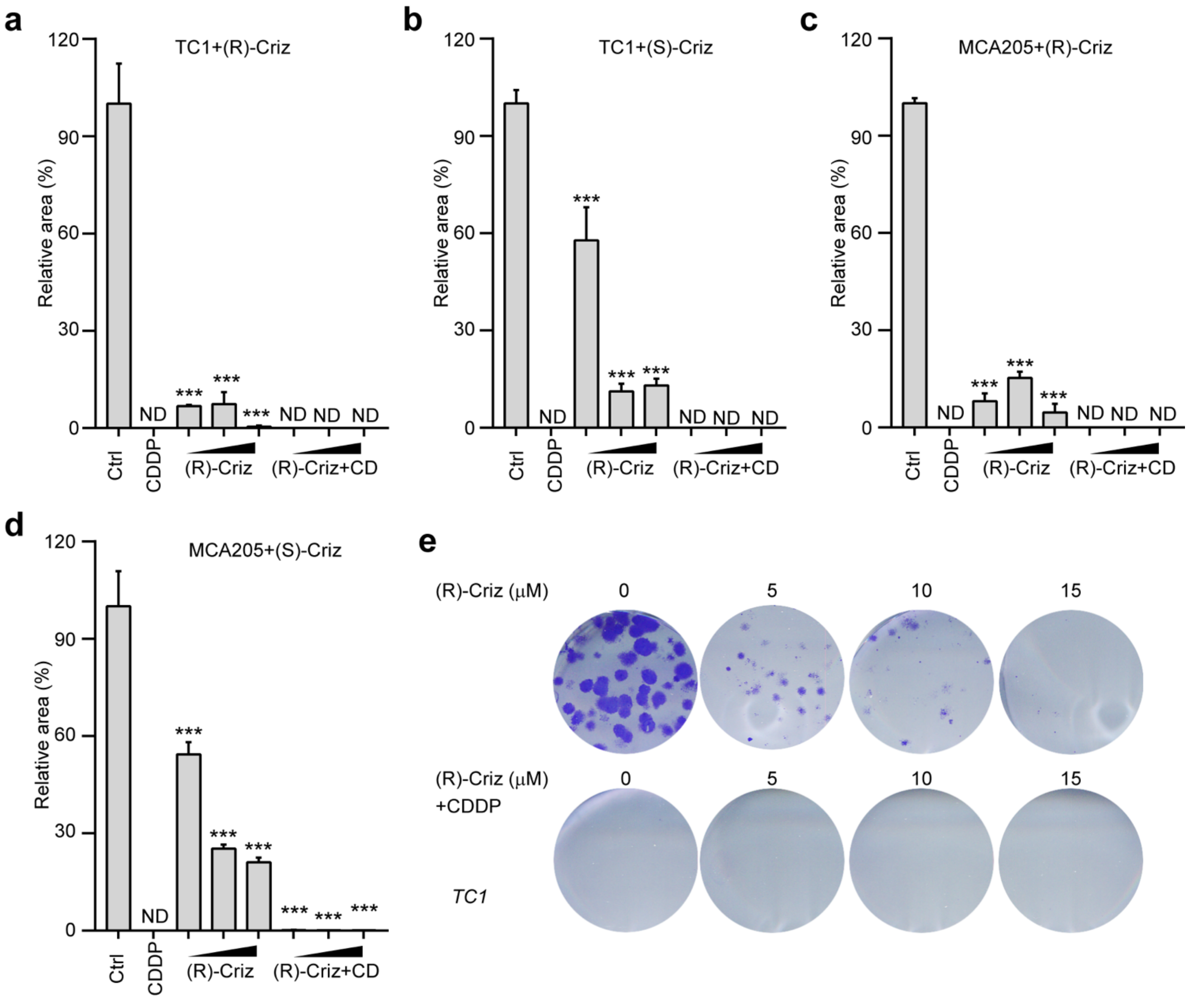

Supplementary Figure 4. Influence of crizotinib on the clonogenicity of mouse tumor cells. TC1 or MCA205 cells were seeded in 6-well plates (200 cells per well) and incubated with (R) or (S)-crizotinib (Criz), cisplatin (CDDP), or their combination (Criz + CD) at the indicated concentrations for 24 h. Then the supernatant was replaced with fresh media. Cells were cultured for 12 days before fixation and staining with crystal violet. Colony areas were quantified by digital analysis using the Image-J software and normalized to values obtained in untreated controls (Ctrl) (**a-d,** mean ± s.e.m., n = 3, ***p < 0.001, compared to Ctrl, t-test, ND = non-detected). Representative images obtained from TC1 cells are shown (**e**).

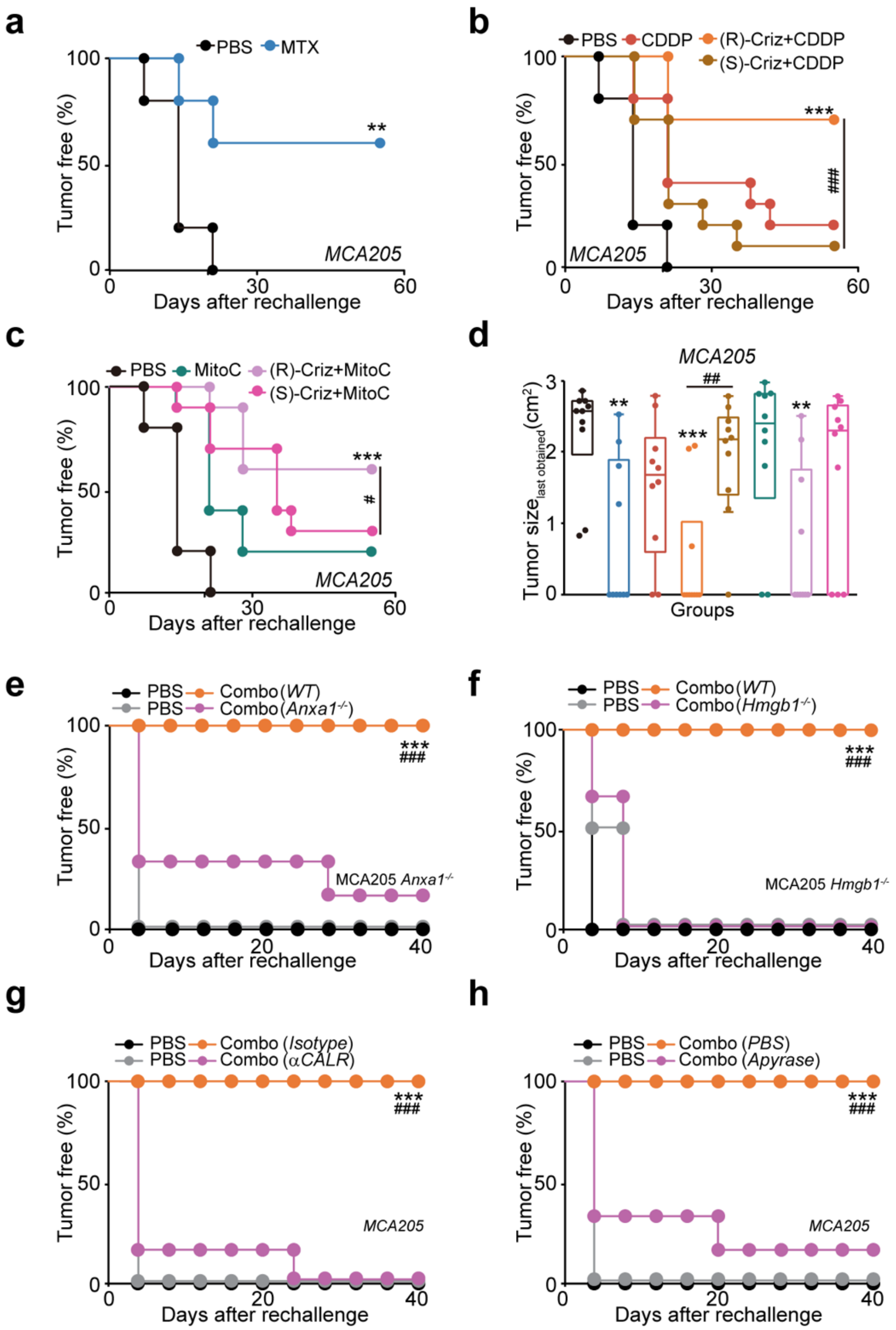

Supplementary Figure 5. (R)-crizotinib had anti-cancer vaccination effects on MCA205 cancer. (**a-d**), Wild type (*WT*) MCA205 cells were treated with mitoxantrone (MTX), cisplatin (CDDP), mitomycin C (MitoC) alone or in combination with (R)- or (S)-crizotinib (Criz) for 24 h, and subcutaneously injected into C57BL/6 mice. Mice were rechallenged with living MCA205 cells 2 weeks later. Tumor incidence over time is reported as Kaplan–Meier curves (**a-c**). Final tumor size distribution at endpoint is shown in (**d**). (**e,f**) Vaccination test with Anxa1-deficient (*Anxa1*$^{-/-}$) or Hmgb1-deficient (*Hmgb1*$^{-/-}$) MCA205 cells following the treatment of (R)-Criz plus CDDP (Combo). (**g,h**) Combo-treated MCA205 cells were incubated with CALR antibody (αCALR) or isotype antibody, or treated with apyrase before injected for vaccination. Statistical significance was calculated by Likelihood ratio test for tumor-free and ANOVA test for tumor size distribution. $^{**}p < 0.01$, $^{***}p < 0.001$ compared to PBS; $^{\#}p < 0.05$, $^{\#\#\#}p < 0.001$ compared between indicated groups. $n = 10$ animals per group.

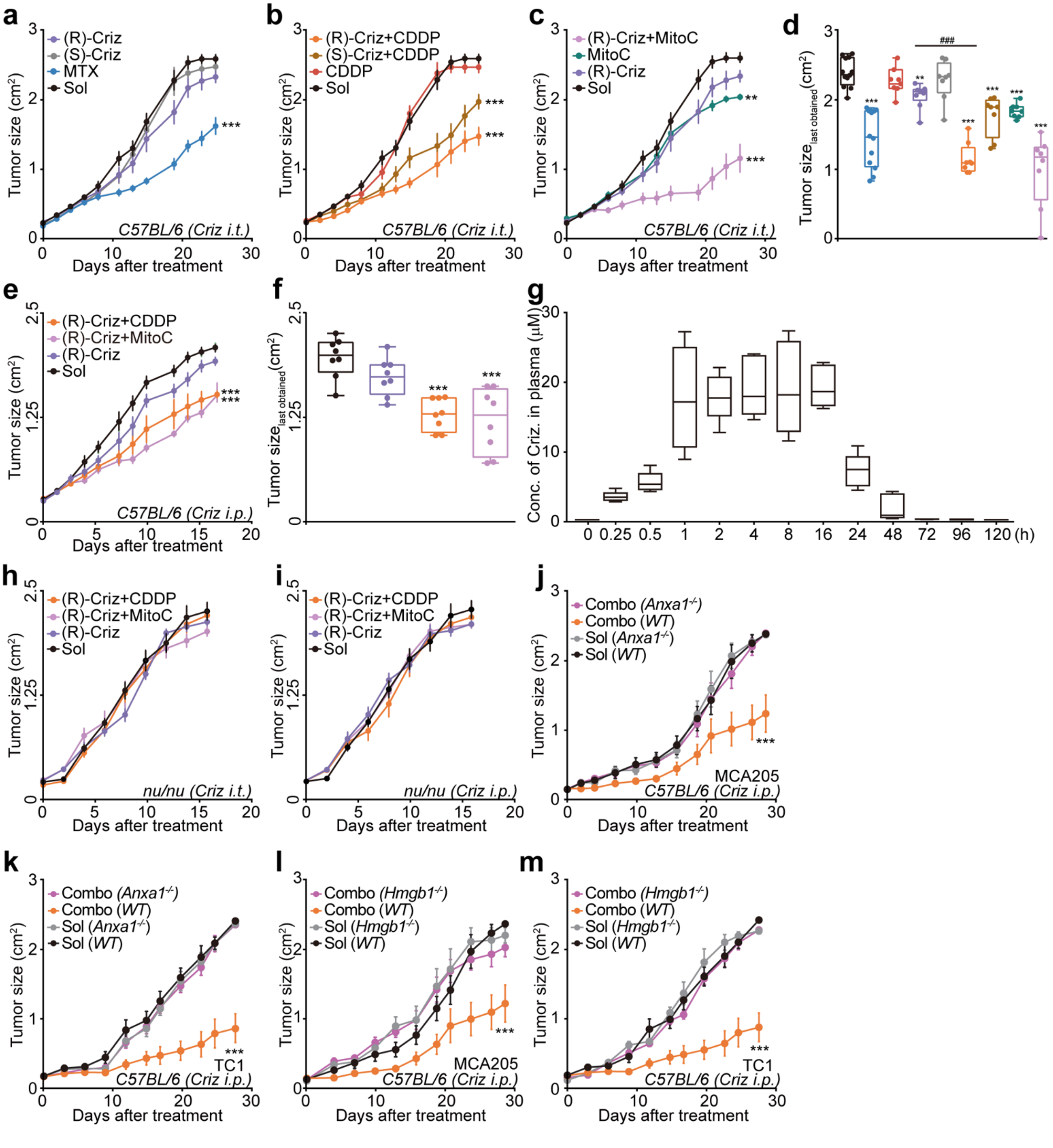

Supplementary Figure 6. (R)-crizotinib in combination with CDDP or MitoC has synergistic and immune dependent anti-cancer effects in subcutaneous cancer models. Living MCA205 cells (5×10^5 per mouse) were subcutaneously injected into the right flank of C57BL/6 (**a-f**) or *nu/nu* mice (**h,i**). When tumors became palpable (day 0), mice received intraperitoneal (*i.p.*) injections of solvent control (Sol), mitoxantrone (MTX), cisplatin (CDDP), mitomycin C (MitoC) alone or in combination with either intertumoral (*i.t.*) or *i.p.* injections of (R)- or (S)-crizotinib (Criz) at day 0 and day 2. Average (mean ± s.e.m.) tumor growth curves (**a-c,e,h,i**) as well as tumor size distribution at endpoint (**d,f**) are reported. Statistical significance was calculated by means of the ANOVA Type 2 (Wald test) for tumor

growth curves, or ANOVA test for multiple comparisons for tumor size distribution. *ns*, non-significant; $^{**}p < 0.01$, $^{***}p < 0.001$ as compared to Sol. $^{###}p < 0.001$ as compared between indicated groups; minimum of $n = 8$ animals per group. (**g**) Quantification of (R)-Criz concentration in mice plasma following an *i.p.* injection. Tumor formed by *Anxa1*$^{-/-}$ (**j,k**) or *Hmgb1*$^{-/-}$ (**l,m**) MCA205 and TC1 cells were compared with WT tumor with respect to their response to treatment with (R)-Criz plus CDDP (Combo, *i.p.*). Representative experiments involving at least six mice per group are reported as mean ± s.e.m. over time. *ns*, non-significant; *** $p < 0.001$ (Wald test), as compared to Sol.

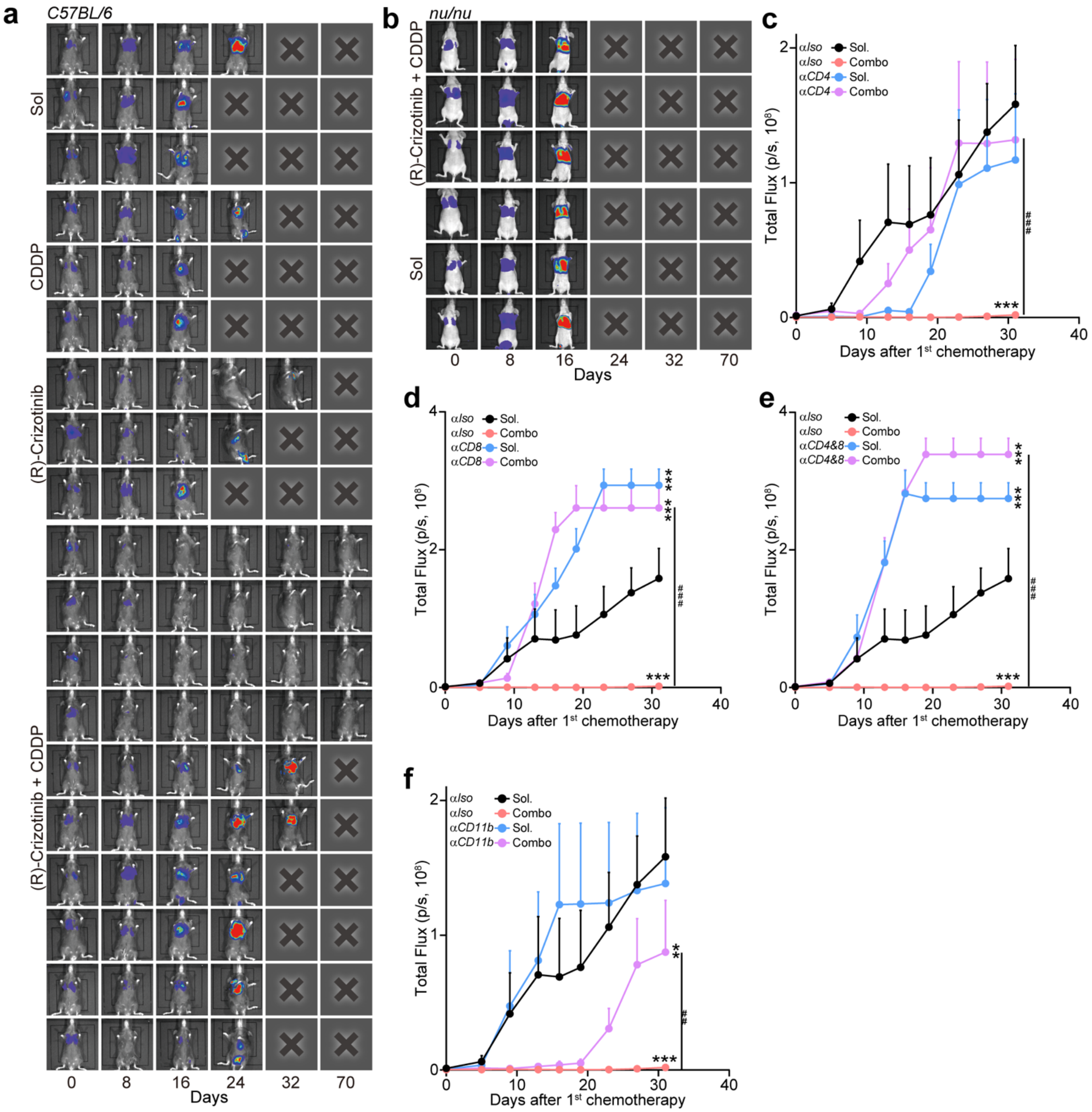

Supplementary Figure 7. (R)-crizotinib plus CDDP mediated tumor growth suppression of orthotopic TC1 lung cancer is T cell-dependent. (**a,b**), representative bioluminescence photographs of orthotopic TC1 cancer in C57BL/6 mice (**a**) and immunodeficient *nu/nu* mice (**b**) upon treatment with (R)-crizotinib plus cisplatin (CDDP) (Combo). (**c-f**) Orthotopic TC1 lung cancers were established and treated with the Combo regimen. For T cell depletion (**c-e**), CD4 and/or CD8-specific antibodies (αCD4, αCD8) were administrated weekly. For inhibiting the extravasation of myeloid cells (**f**), a CD11b-specific antibody (αCD11b) was administrated every other day. Tumor sizes were quantified as total flux of the photons and summarized as mean ± s.e.m. over time (*n* = 8 animals per group), Wald test, *** $p < 0.001$ as compared to solvent control (Sol); $^{\#\#}p < 0.01$, $^{\#\#\#}p < 0.001$ compared between indicated groups.

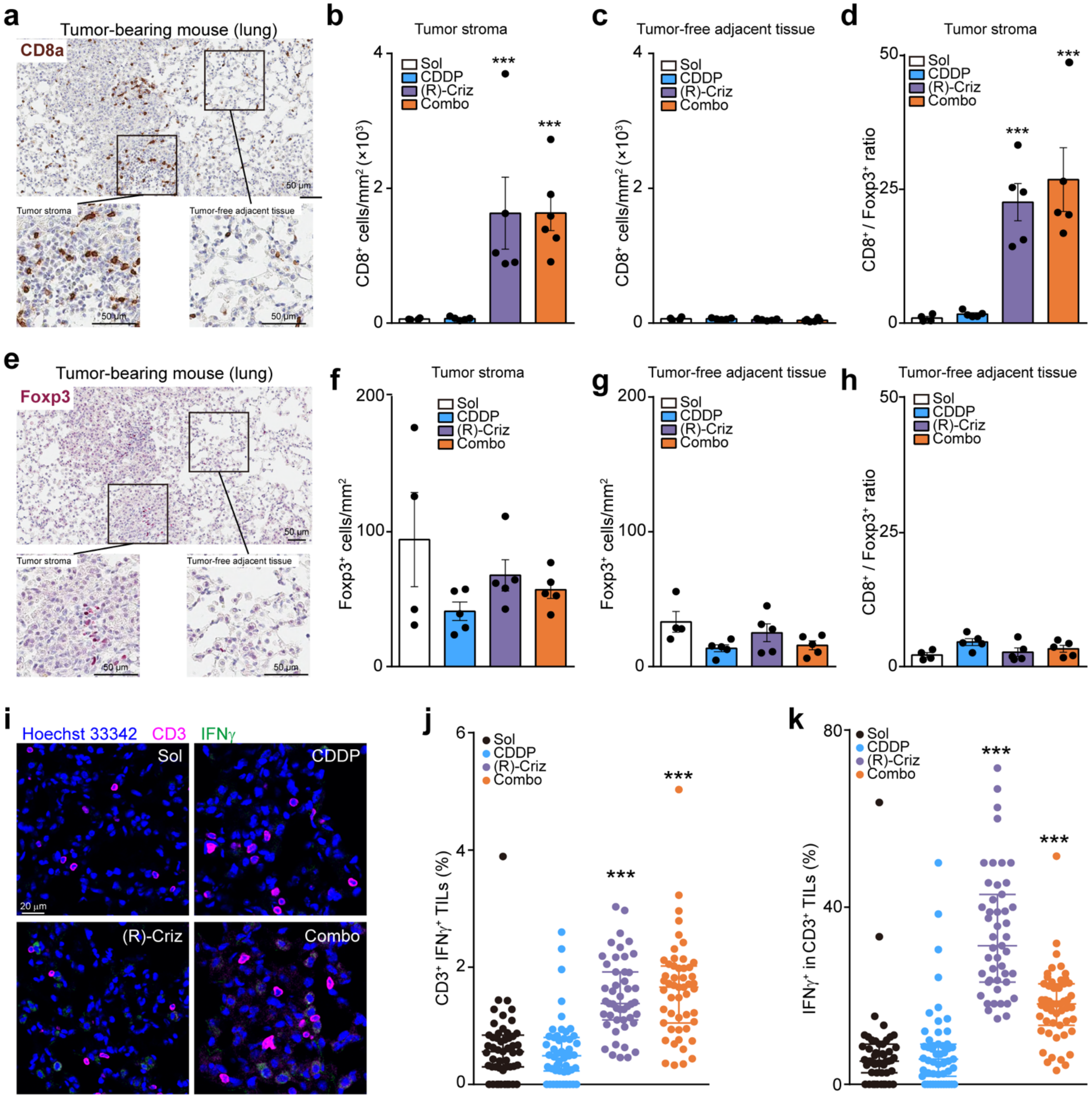

Supplementary Figure 8. **(R)-crizotinib changed the immune infiltrate in KP and urethane induced lung cancers.** Lung lobes from the KP- and urethane-lung cancer-bearing models were harvested and cut for IHC staining following the treatment of solvent (Sol), (R)-crizotinib (Criz), cisplatin (CDDP) or their combination (Combo). In the KP model, cytotoxic $CD8^+$ T cells (**a-c**) and regulatory $Foxp3^+$ cells (Treg) (**e-g**) were quantified in both tumor and normal lung tissues. Representative stained sections containing tumor and tumor-free adjacent tissues are presented (**a,e**). The $CD8^+$ T cell to Treg ratio was calculated (**d,h**). Results are expressed as mean ± s.e.m.. ***$p <$ 0.001 as compared to Sol, *t*-test, *n* = minimum of 4 mice per group. In the urethane induced lung cancer model, frozen lung lobes were subjected to immunofluorescence staining of CD3 and IFN-γ. Representative images are shown in (**i**), and quantified images are reported as scatter dot plots (median with interquartile range) (**j,k**). ***$p < 0.001$ as compared to Sol, two-way ANOVA test, *n* = minimum of 9 mice per group.

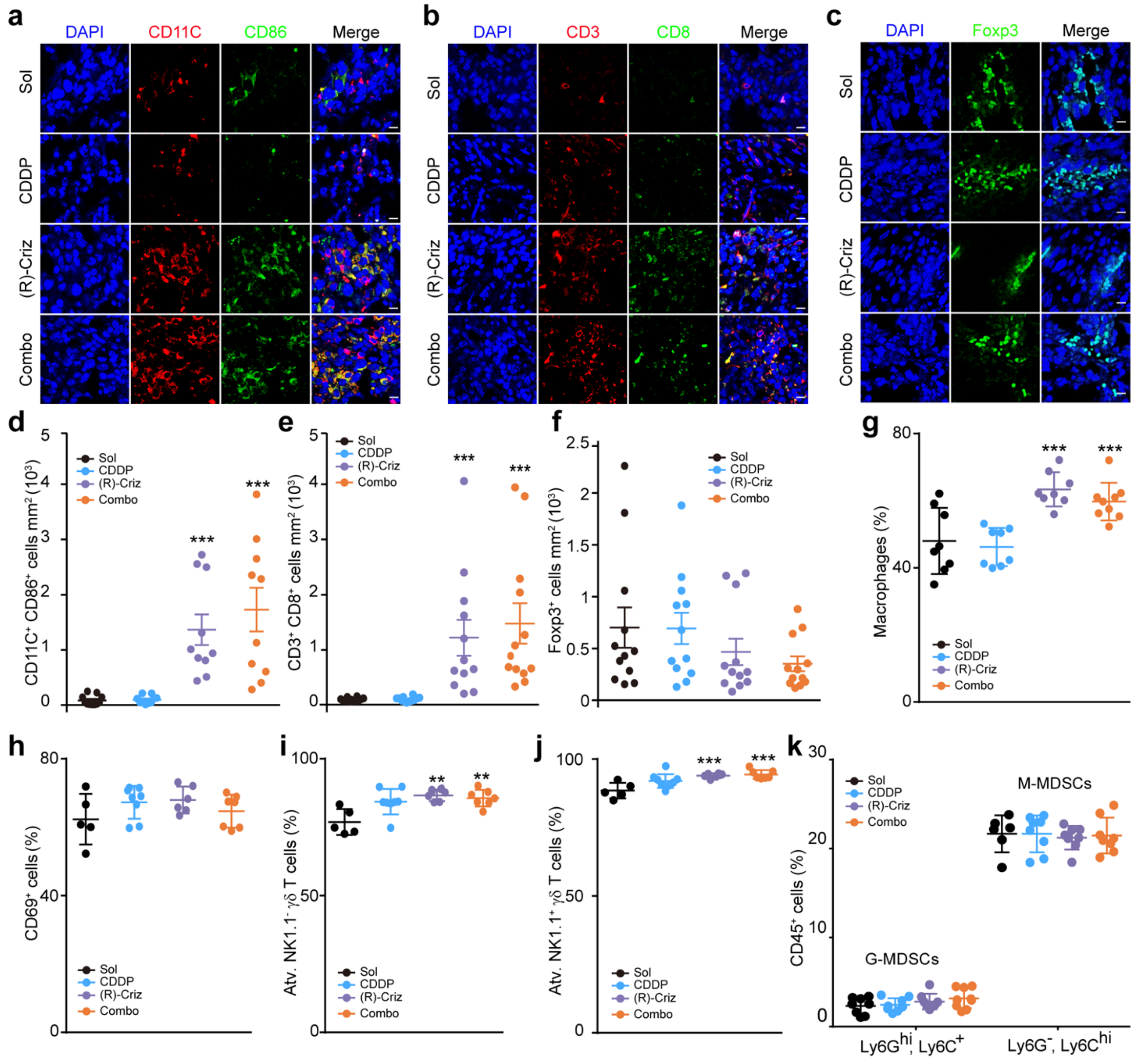

Supplementary Figure 9. (R)-crizotinib changed immune infiltration in subcutaneous tumors. (a-f) C57BL/6 mice bearing MCA205 tumors were treated with solvent (Sol), cisplatin (CDDP), (R)-crizotinib (Criz) or their combination (Combo). Tumors were harvested at day 8 posts 1st treatment, and 5 µm sections were subjected to immunofluorescence staining. Representative images and quantifications of tumor-infiltrating CD11c^{+} CD86^{+} mature dendritic cells (**a, d**), bulk of CD3^{+} CD8^{+} cytotoxic T cells (**b,e**), and Foxp3^{+} cells (**c,f**) are depicted, ***$p < 0.001$ as compared to Sol (ANOVA test, n = minimum of 10 mice per group), scale bar equals 10 µm. Alternatively, after the same treatment tumors were harvested at day 10 and digested to single cell suspensions for cytofluorimetric analyses. Inflammatory macrophages are displayed as % of F4-80^{+} MHCII^{hi} among Ly6G^{-} Ly6C^{hi} CD11c^{hi} CD11b^{+} leukocytes (**g**); total activated NK cells are displayed as % of CD69^{+} cells among CD3^{-} NK 1.1^{+} TCRγδ^{-} cells (**h**); activated γδ T cells are displayed as % of CD69^{+} cells among NK1.1^{-} (panel **i**) or NK1.1^{+} (panel **j**) TCRγδ^{+} CD3^{+} lymphocytes; Ly6G^{hi}Ly6C^{+} (G-MDSC) and Ly6G^{-}Ly6C^{hi} (M-MDSC) populations are expressed as % of CD45^{+} leukocytes (**k**). **$p < 0.01$, ***$p < 0.001$ as compared to Sol (ANOVA test, n = minimum of 6 mice per group).

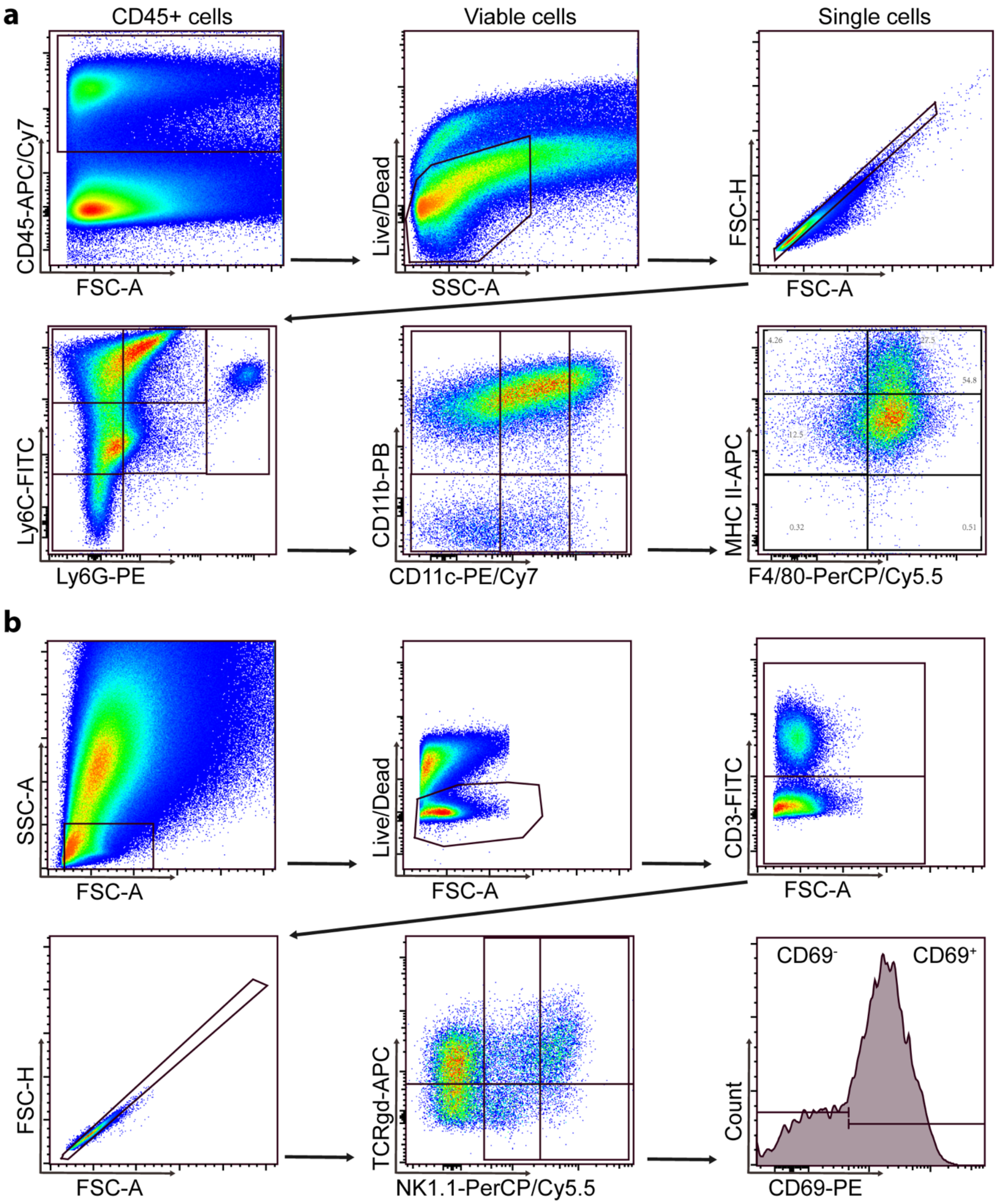

Supplementary Figure 10. Gating strategy for the flow cytometric analysis of tumor-infiltrating myeloid cells and NK cells. (**a**) Myeloid cell gating strategy. Cells were first gated on FSC and SSC, followed by gating on $CD45^{+}$ leukocytes, gating on life cells (by Live/Dead dye) and exclusion of doublets. Live leukocytes were hierarchically sub-gated according to the expression levels of Ly6C/Ly6G and CD11c/CD11b to describe different myeloid cell populations. Specific myeloid cell types were identified based on the expression of MHC II and F4/80 to characterize macrophages. (**b**) Phenotype of NK-like cells. Initial gating was performed to exclude non-lymphoid cells based on FSC and SSC, and the Live/Dead dye to exclude dead cells. Doublets were excluded, and CD3 expression was used as a T cell marker. NK cells and γδT cells can be discriminated according to CD3/NK1.1/γδTCR expression. CD69 was considered as an activation marker.

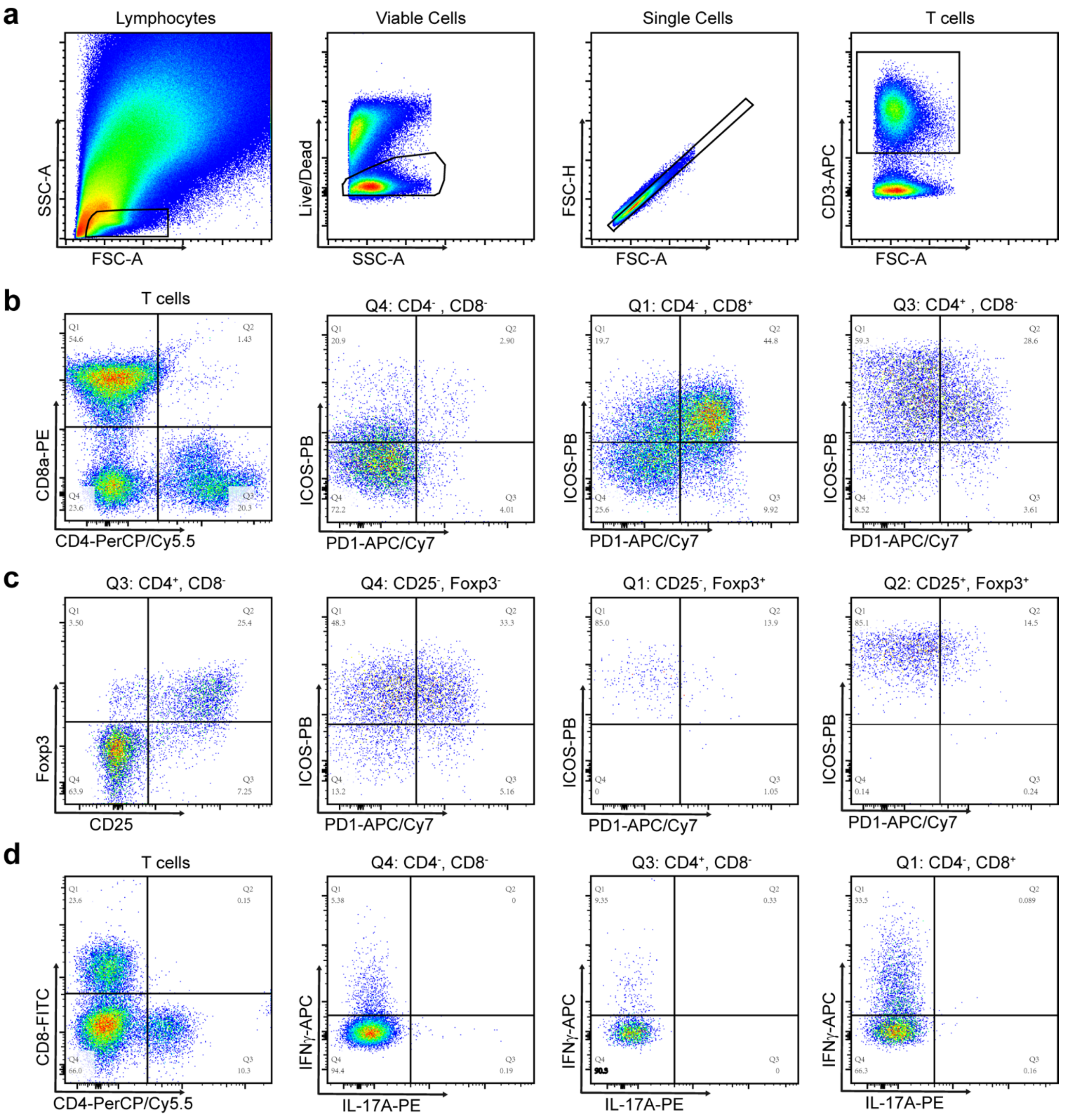

Supplementary Figure 11. Gating strategy for the flow cytometric analysis of tumor-infiltrating T cells. Initial gating was performed to first exclude non-lymphocyte cells based on FSC and SSC. LIVE/DEAD® fixable dye was used to discriminate viable from damaged/dead cells. Doublets were excluded and $CD3^{+}$ cells were gated among singlets (**a**). $CD3^{+}$ cells were then separated into $CD8^{+}$ or $CD4^{+}$ cells while excluding double positive auto-fluorescent cells for further T cell subpopulation profiling based on the expression of ICOS, PD-1 and CTLA4 (**b**). Intracellular Foxp3 expression within the CD4 positive population allowed to discriminate regulatory T cells (Tregs) from non-Tregs (**c**), while expression of IFNγ and IL-17 allowed to identify Th1 and Th17 cells, respectively (**d**).

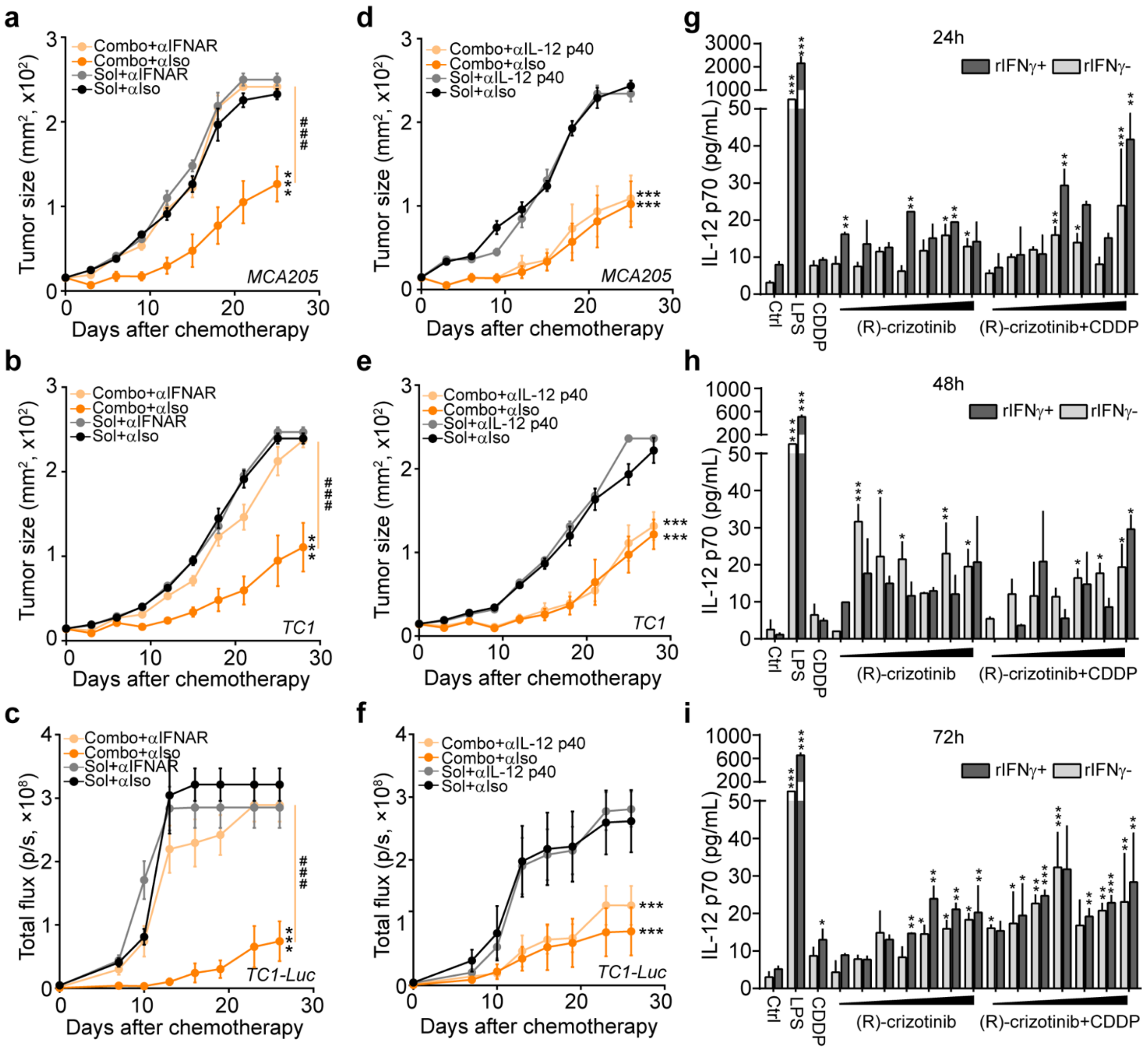

Supplementary Figure 12. (R)-crizotinib plus CDDP mediated tumor growth suppression is dependent on IFNγ production. C57BL/6 mice bearing subcutaneous MCA205 (**a,d**), TC1 (**b,e**), or orthotopic TC1 *Luc* tumors (**c,f**) were treated with solvent control (Sol) or (R)-crizotinib plus cisplatin (CDDP) (Combo) as described above, accompanied by the administration of antibodies neutralizing IFNAR-1(αIFNAR) or IL-12 p40 (αIL-12 p40) or the corresponding isotype antibodies (αIso). Tumor development was monitored in all models, and statistical significance was calculated by means of the ANOVA Type 2 (Wald test) for tumor growth curves. ***p < 0.001 compared to Sol + αIso, $^{###}$p < 0.001 comparing the indicated groups. (**g-i**) IL-12 secretion by bone marrow-derived dendritic cells following the treatment of (R)-crizotinib (0.1, 0.25, 0.5, 1, 2.5, 5, 10 μM) alone or in combination with CDDP for different periods, either or not pretreated by recombinant IFNγ (rIFNγ), was measured by ELISA. LPS was used as a positive control. Data are shown as mean ± s.e.m. (n = 3; *p < 0.05, **p < 0.01, ***p < 0.001, compared to untreated control (Ctrl), t-test).

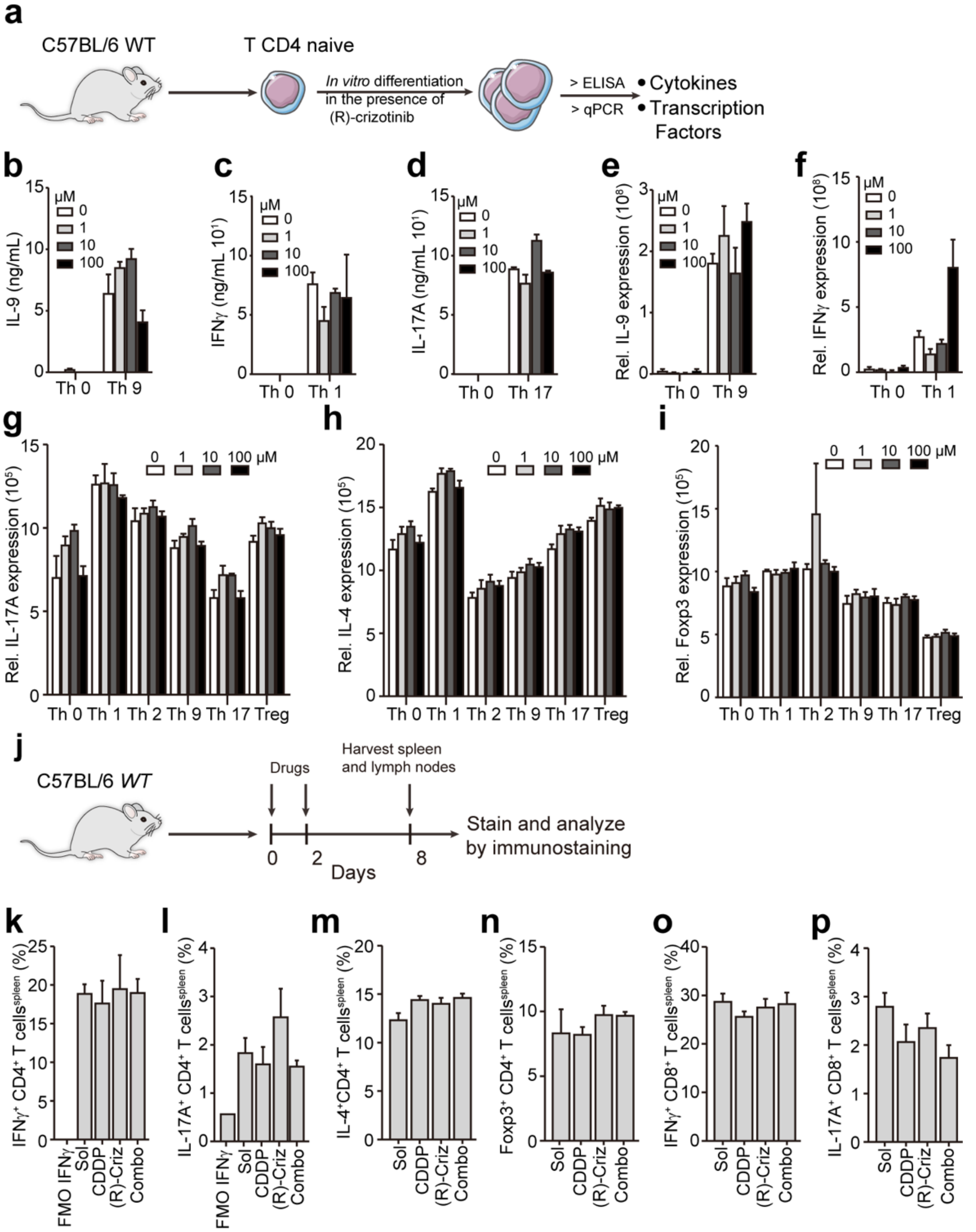

Supplementary Figure 13. (R)-crizotinib alone or in combination with cisplatin showed no effect on the immune system of naïve mice. *In vitro* differentiation of T-lymphocytes subtypes (**a**) was measured by assessing the indicated markers by means of ELISA (**b-d**) or real-time PCR (**e-i**) following the treatment of (R)-crizotinib (Criz) at different concentrations. Naïve mice were treated with solvent (Sol), cisplatin (CDDP), (R)-Criz, or their combination (Combo), and *in vivo* immune analyses were performed according to the scheme reported in (**b**). The frequency of IFNγ, IL-4 or IL-17 producing $CD4^+$ or $CD8^+$ T cells was analyzed by means of PMA-ionomycin stimulation, immunofluorescence staining and cytofluorometric analysis (**k-p**). Results are expressed as mean ± s.e.m.

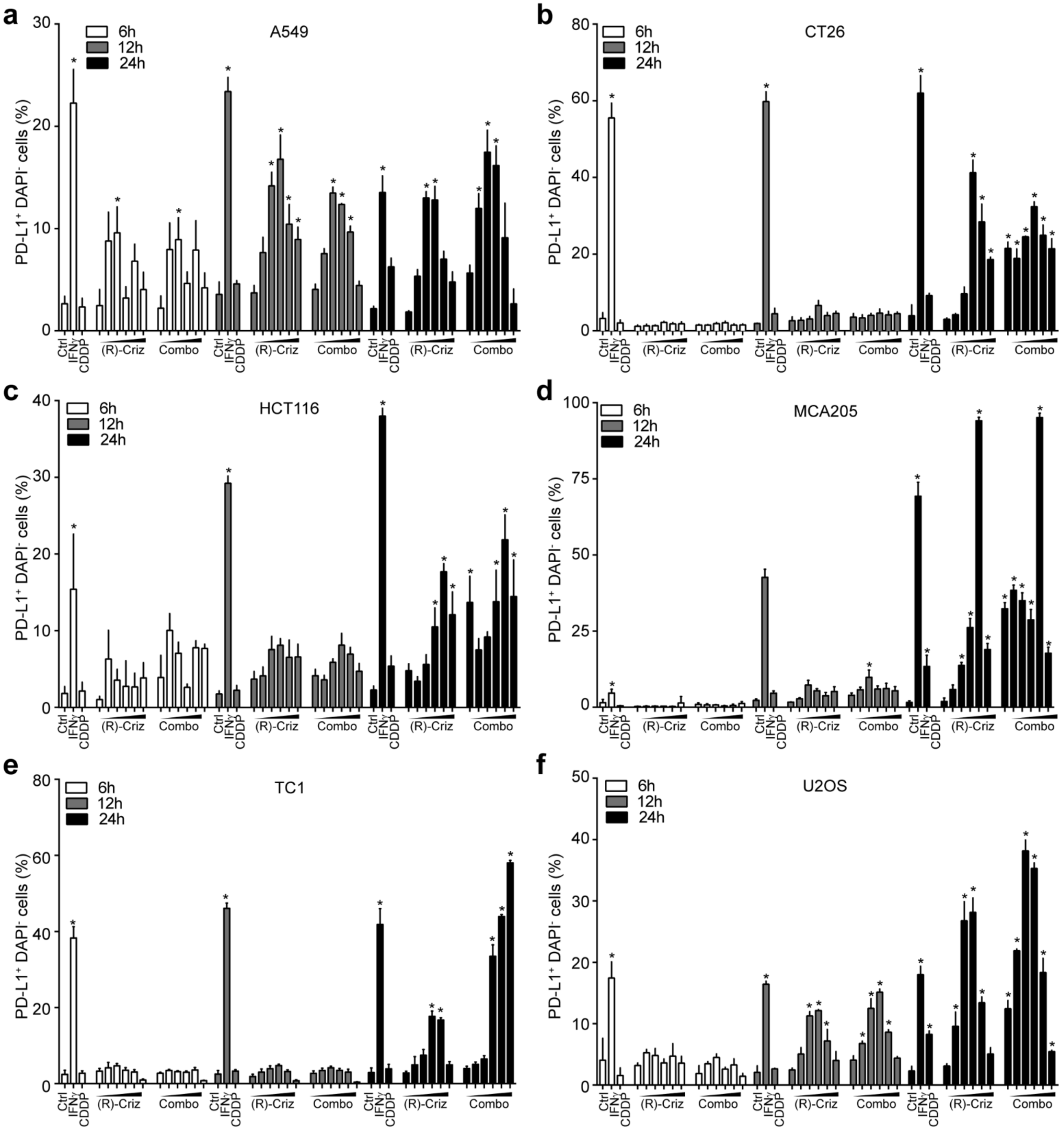

Supplementary Figure 14. (R)-crizotinib in combination with cisplatin induced high expression of PD-L1 on tumor cells. Multiple human (A549, HCT116, U2OS) and murine (CT26, MCA205, TC1) cancer cell lines were treated with increasing concentrations (0.1, 0.25, 0.5, 1, 2.5, 5 μM) of (R)-crizotinib (Criz) alone or in combination with cisplatin (CDDP) (Combo) for 6, 12, or 24 hours before surface staining with PD-L1-specific antibodies. Stimulation with recombinant murine IFNγ was used as a positive control. Data are shown as mean ± s.e.m. (*n* = 3; ***p < 0.001, compared to untreated controls (Ctrl) using Student's t-test).

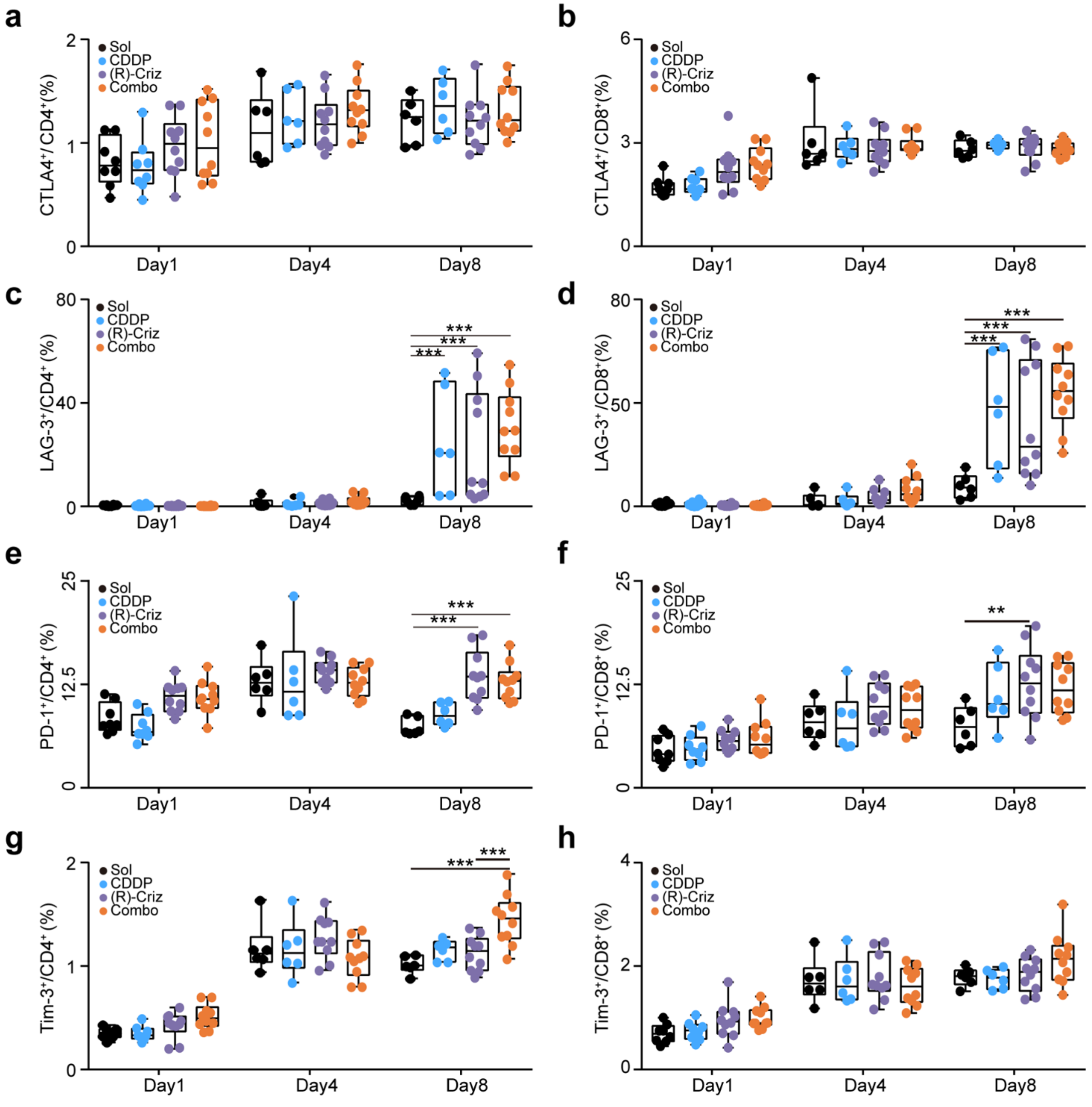

Supplementary Figure 15. (R)-crizotinib plus cisplatin changes the expression of T cell exhaustion markers. C56BL/6 mice bearing MCA205 tumors were treated with solvent (Sol), (R)-Crizotinib (Criz), cisplatin (CDDP), or their combination (Combo) at day 0 (when tumors became palpable) and day 2. Whole blood was collected at day 8 for isolation of PBMCs, which were subjected to immunostaining of different T cell exhaustion markers. The percentages of CTLA-4 (**a,b**) LAG-3(**c,d**), PD-1(**e,f**), and TIM-3 (**g,h**) positive cells among $CD4^+$ and $CD8^+$ T cell populations are depicted as box plots with individual sample points. Statistical significance is shown as **P<0.01, ***P<0.001 (two-way ANOVA using Dunnett's multiple comparisons test), *n* = minimum of 6 animals per group.

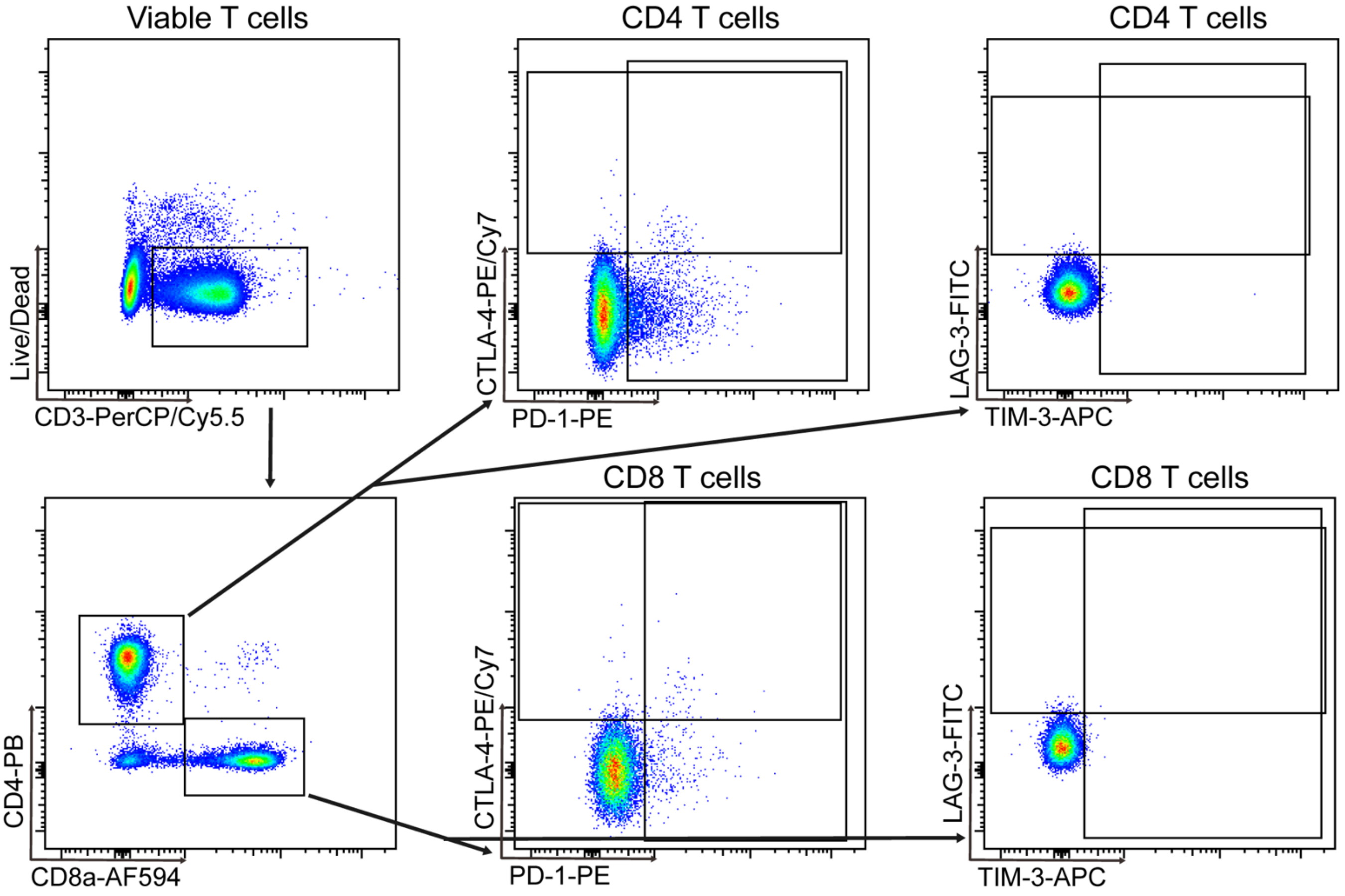

Supplementary Figure 16. Representative flow cytometry gating strategy for T cell exhaustion markers analysis. Initial gating was performed to exclude non-lymphoid cells based on the FSC and SSC characteristics. Live-Dead^{-} and CD3^{+} were gated on to focus on viable T cells. CD3^{+} cells were further separated into CD8^{+} or CD4^{+} cells. CD4^{+} or CD8^{+} cells carrying the indicated exhaustion markers were then identified.

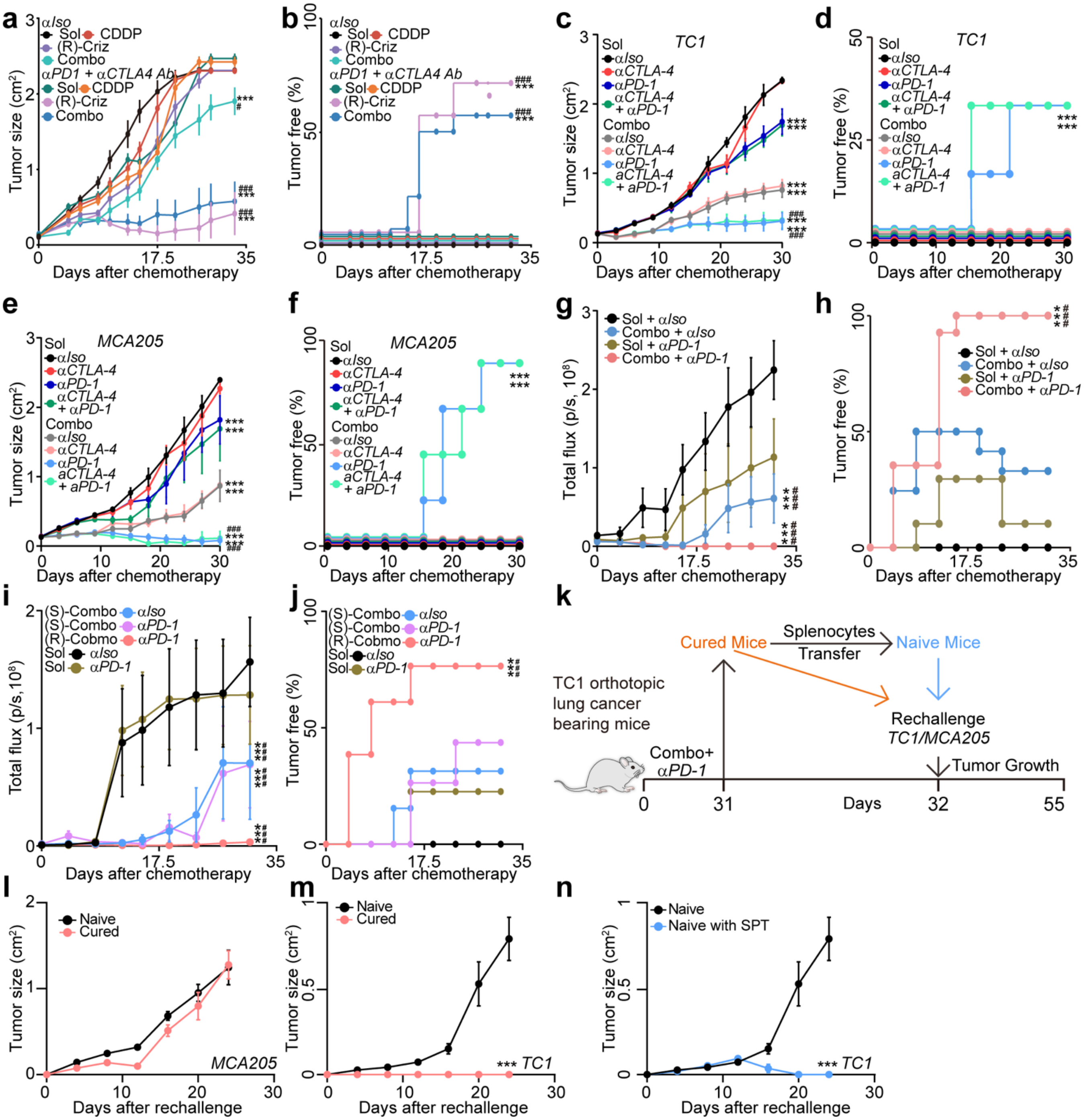

Supplementary Figure 17. (R)-crizotinib sensitizes tumors to checkpoint blockade in established cancers. C57BL/6 mice bearing subcutaneous (*s.c.*) MCA205 tumors (**a,b,e,f**), *s.c.* TC1 tumors (**c,d**), orthotopic LLC1 *Luc* lung cancers (**g,h**), as well as orthotopic TC1 *Luc* lung cancers (**i,j**) received treatments with solvent (Sol), cisplatin (CDDP), (R)/(S)-crizotinib (Criz), or their combinations (Combo, or (S)-Combo when using (S)-Criz) plus monoclonal antibodies specific for PD-1 (αPD-1) and/or CTLA-4 (αCTLA-4), or equivalent isotype control antibodies (αIso) according to the scheme in **Fig. 7**. Average tumor growth (**a,c,e,g,i**) and the percentage of tumor free animals (**b,d,f,h,j**) are reported. Statistical significance was calculated by means of the ANOVA type 2 (Wald test) for tumor growth curves, and likelihood ratio test for tumor free. $^{***}p < 0.001$ as compared to Sol + αIso; $^{###}p < 0.001$ as compared to Sol + αPD-1 + αCTLA-4 (**a-f**), or to Sol + αPD-1 (**g-j**), n = minimum 6 animals per group. Mice cured from orthotopic TC1 *Luc* lung cancers were used as donors of splenocytes that were

adoptively transferred into naïve mice. Then cured mice (positive control), splenocytes transferred (SPT) naïve mice, and PBS-injected naïve mice (negative control) were rechallenged by s.c. injection of TC1 *Luc* and MCA205 cells into two opposite flanks (**k**). The growth of both tumor types is reported in (**l-n**).

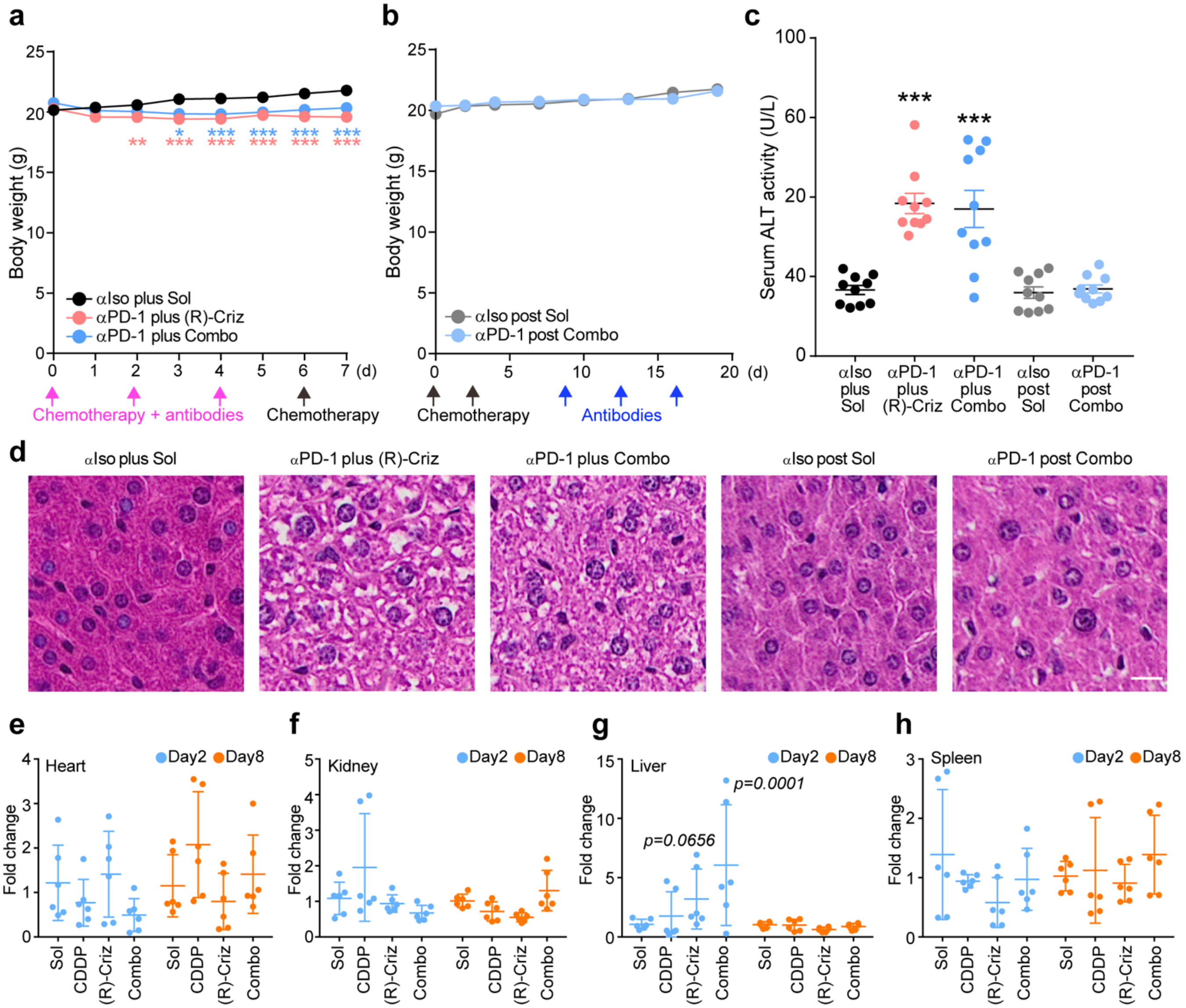

Supplementary Figure 18. Hepatotoxicity induced by simultaneous but not sequential administrations of (R)-crizotinib and anti-PD-1. MCA205 tumor bearing mice received simultaneous PD-1 antibody (αPD-1) plus solvent (Sol), (R)-crizotinib (Criz) alone or in combination with cisplatin (CDDP) (Combo), or a sequential regime of αPD-1 post the Combo treatment, as illustrated in (**a**) and (**b**), during which bodyweight was recorded daily (*P<0.05, **P<0.01, ***P<0.001, t test). Serum alanine transaminase (ALT) activity was measured at day 3 after last αPD-1 injection (**c**). ***p < 0.001 (two-way ANOVA, Dunnett's test) as compared to mice received only Sol + isotype antibody (αIso). Livers were fixed for HE staining revealing cytoplasmic vacuolization of hepatocytes in mice receiving concomitant treatment with Criz or Combo plus αPD-1. The scale bar equals 10 μm (**d**). Alternatively, mouse organs were recovered at day 2 and day 8 after the indicated treatments and subjected to mRNA extraction for qPCR analysis of *PD-L1* expressions (**e-h**). *P* value is annotated (**g**) based on the two-way ANOVA test.

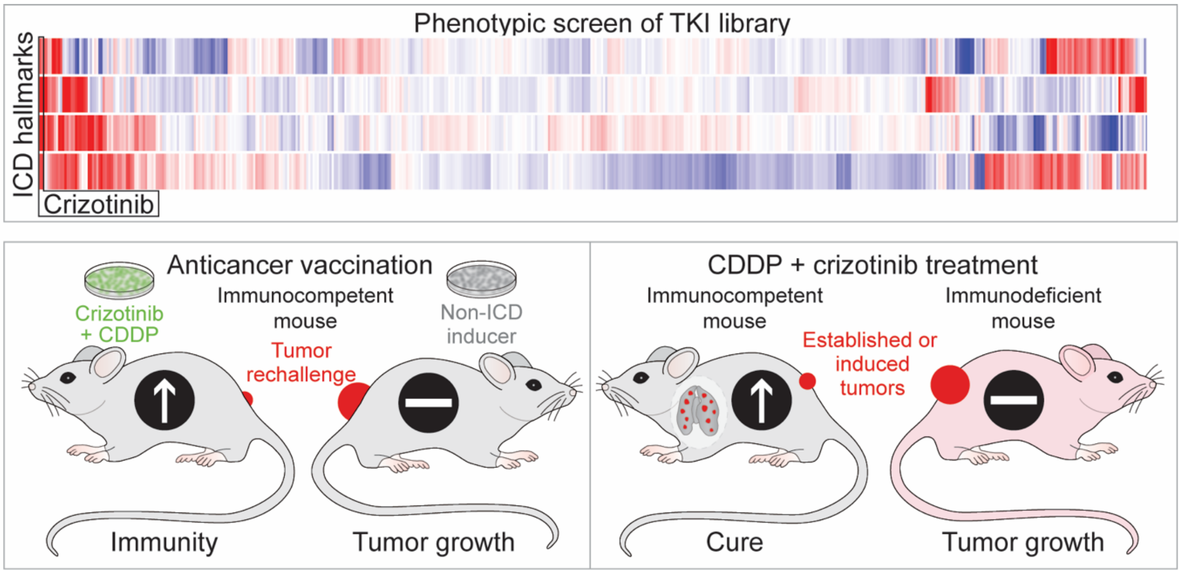

Supplementary Figure 19. Graphical abstract of this study

Supplementary table 1. Primers for qPCR

Gene	species	Forward sequence	Reverse sequence
HPRT1	Human	CCTGGCGTCGTGATTAGTGA	CGAGCAAGACGTTCAGTCCT
Gapdh	Mouse	CGACTTCAACAGCAACTCCCACT CTTCC	TGGGTGGTCCAGGGTTTCTTACTCCTT
IFNα1	Human	ACTCATACACCAGGTCACGC	CAGTGTAAAGGTGCACATGACG
Ifnα1	Mouse	TTTCCCCTGACCCAGGAAGATG	CTCTCAGTCTTCCCAGCACATT
IFNα2	Human	TGATTCGTATGCCAGCTCACC	TCAGTCAGCATGGTCCTCTGT
Ifnα2	Mouse	TACTCAGCAGACCTTGAACCT	CAGTCTTGGCAGCAAGTTGAC
IFNβ1	Human	AGTAGGCGACACTGTTCGTG	GCCTCCCATTCAATTGCCAC
Ifnβ1	Mouse	GTCCTCAACTGCTCTCCACT	CCTGCAACCACCACTCATTC
ALK	Human	ACCCTGAAAGCCACAAGGTC	TTCCGGCGGTACACAATCAT
Alk	Mouse	ACTGACATCCTCGCTTCTGAA	ATACGTTTCCTCTCAAAACCCC
JAK2	Human	CCGATCTGTGTAGCCGGTTT	GTAAGGCAGGCCATTCCCAT
Jak2	Mouse	TTGTGGTATTACGCCTGTGTATC	ATGCCTGGTTGACTCGTCTAT
MET	Human	AGCAATGGGGAGTGTAAAGAGG	CCCAGTCTTGTACTCAGCAAC
Met	Mouse	GTGAACATGAAGTATCAGCTCCC	TGTAGTTTGTGGCTCCGAGAT
ROS1	Human	ATGCAGACCTACCAACTGCTC	GGGCTTGACCACATAGGACG
Ros1	Mouse	CTGCCTAACGTCCTGTGTAAC	CAGAGTTCCAAAACTGACATCCA
Ctla4	Mouse	CATGGTGTCGCCAGCTTTC	GGTAATCTAGGAAGCCCACTGTA
Pd1	Mouse	ACCCTGGTCATTCACTTGGG	CATTTGCTCCCTCTGACACTG
Pdl1	Mouse	CACTTGCTACGGGCGTTTAC	CCCAGTACACCACTAACGCA
Actb	Mouse	ATGGAGGGGAATACAGCCC	TTCTTTGCAGCTCCTTCGTT
Ifng	Mouse	GAGCTCATTGAATGCTTGGC	GCGTCATTGAATCACACCTG
Il4	Mouse	CGAGCTCACTCTCTGTGGTG	TGAACGAGGTCACAGGAGAA
Il9	Mouse	AACAGTCCCTCCCTGTAGCA	AAGGATGATCCACCGTCAAA
Il17a	Mouse	TGAGCTTCCCAGATCACAGA	TCCAGAAGGCCCTCAGACTA
Foxp3	Mouse	CTCGTCTGAAGGCAGAGTCA	TGGCAGAGAGGTATTGAGGG
