# Supplementary material for: Crizotinib-induced immunogenic cell death in non-small cell lung cancer
Source: Nat Commun. 2019 Apr 2;10:1486. doi: 10.1038/s41467-019-09415-3 (PMC6445096; doi:10.1038/s41467-019-09415-3)
Supplement: Supplementary file 3 — Reporting Summary [file 41467_2019_9415_MOESM3_ESM.pdf]

nature research

Corresponding author(s): Guido Kroemer

Reporting Summary

Nature Research wishes to improve the reproducibility of the work that we publish. This form provides structure for consistency and transparency in reporting. For further information on Nature Research policies, see Authors & Referees and the Editorial Policy Checklist.

Statistical parameters

When statistical analyses are reported, confirm that the following items are present in the relevant location (e.g. figure legend, table legend, main text, or Methods section).

n/a	Confirmed	
☐	☒	The exact sample size (n) for each experimental group/condition, given as a discrete number and unit of measurement
☐	☒	An indication of whether measurements were taken from distinct samples or whether the same sample was measured repeatedly
☐	☒	The statistical test(s) used AND whether they are one- or two-sided<br>*Only common tests should be described solely by name; describe more complex techniques in the Methods section.*
☒	☐	A description of all covariates tested
☐	☒	A description of any assumptions or corrections, such as tests of normality and adjustment for multiple comparisons
☐	☒	A full description of the statistics including central tendency (e.g. means) or other basic estimates (e.g. regression coefficient) AND variation (e.g. standard deviation) or associated estimates of uncertainty (e.g. confidence intervals)
☐	☒	For null hypothesis testing, the test statistic (e.g. F, t, r) with confidence intervals, effect sizes, degrees of freedom and P value noted<br>*Give P values as exact values whenever suitable.*
☒	☐	For Bayesian analysis, information on the choice of priors and Markov chain Monte Carlo settings
☒	☐	For hierarchical and complex designs, identification of the appropriate level for tests and full reporting of outcomes
☒	☐	Estimates of effect sizes (e.g. Cohen's d, Pearson's r), indicating how they were calculated
☐	☒	Clearly defined error bars<br>*State explicitly what error bars represent (e.g. SD, SE, CI)*

Our web collection on statistics for biologists may be useful.

Software and code

Policy information about availability of computer code

Data collection	Fluorescent images were collected by using a robot-assisted Molecular Devices IXM XL BioImager (Molecular Devices) equipped with a Sola light source (Lumencor), adequate excitation and emission filters (Semrock) a 16-bit monochrome sCMOS PCO.edge 5.5 camera (PCO) and a 20 X PlanAPO objective (Nikon); Flow cytometry data was obtained on a LSRFortessa (BD Biosciences) or on a CyAn ADP cytofluorometer (Beckman Coulter); Luminescence and absorbance were measured using a SpectraMax I3 multi-mode microplate reader (Molecular Devices); qPCR data was obtained with a StepOnePlus Real-Time PCR System (Applied Biosystems); Western blotting signal was obtained on a ImageQuant LAS 4000 software-assisted imager (GE Healthcare); Extracellular acidification rate was analyzed by the Wave Desktop software assisted Seahorse XFe96 Analyzer (Agilent Technologies); Mass spectrometry data was obtained with a RRLC 1260 system (Agilent Technologies) coupled to a 6500+ QTRAP MS (Sciex) equipped with an electrospray ion source; In vivo luciferase photons were acquired on a Xenogen IVIS 50 bioluminiscence in vivo imaging system (Caliper Life Sciences Inc.,); Confocol fluorescent images were acquired on a HR-SP8 Confocal Microscope (Leica); Lung lobes of urethane induced model were imaged with a stereo microscope (Motic SMZ168TP) equipped with a high-resolution camera (Sony HD 1/1.8 inch color CCD); Histological images were obtained with the NanoZoomer 2.0-RS slide scanner system (Hamamatsu). Quantification of clonogenicity was obtained with Image J (https://imagej.nih.gov/ij/)
Data analysis	Fluorescent images were processed and segmented with the MetaXpress software (Molecular Devices); FSC files were processed with the FlowJo software (Tree Star); Integration and quantification of the mass spectrometry analytes were performed using the MultiQuant quantitative software (Version 3.0.3); In vivo luciferase photons were quantified with the living Image® software V3.2; stereo microscope images were quantified with the supporting software EZ-NET™; Aperio ImageScope Viewer (Leica Biosystems) was used for histological image quantification; GraphPad Prism 7 (https://www.graphpad.com/), Microsoft Excel 2010, and the freely available software R

(https://www.r-project.org) were used for statistical analysis and graphical data presentation. Tumor growth statistical analysis was performed with the TumGrowth software developed by the lab (https://kroemerlab.shinyapps.io/TumGrowth/).

For manuscripts utilizing custom algorithms or software that are central to the research but not yet described in published literature, software must be made available to editors/reviewers upon request. We strongly encourage code deposition in a community repository (e.g. GitHub). See the Nature Research guidelines for submitting code & software for further information.

Data

Policy information about availability of data

All manuscripts must include a data availability statement. This statement should provide the following information, where applicable:

- Accession codes, unique identifiers, or web links for publicly available datasets
- A list of figures that have associated raw data
- A description of any restrictions on data availability

Supporting data for figure1 a and b can be found in supplementary table 1 and 2; supporting data for figure 6e,f can be found in supplementary table 3; sequences of qPCR primers are reported in the materials and methods part; other datasets supporting the findings of this study are available from the corresponding author upon request.

Field-specific reporting

Please select the best fit for your research. If you are not sure, read the appropriate sections before making your selection.

☒ Life sciences ☐ Behavioural & social sciences ☐ Ecological, evolutionary & environmental sciences

For a reference copy of the document with all sections, see nature.com/authors/policies/ReportingSummary-flat.pdf

Life sciences study design

All studies must disclose on these points even when the disclosure is negative.

Sample size	Sample sizes were decided based on previous publications and experience and common standards in similar field, for calculating statistical significance. Sample sizes and number of independent experiments are always indicated in the figure legends or in the Materials & Methods. For all data subjected to statistical analyses, results from three or more independent repeats were used. Number of animals in all in vivo experiments are stated in the figure legends.
Data exclusions	No data was excluded in experiments that can be used to generate data to support this manuscript.
Replication	All experiments described in this manuscript were reproduced.
Randomization	All cell based image analysis were randomized. Mice of same age and gender were randomized for all in vivo experiments.
Blinding	Quantification of histological images were blinded and independently performed by coauthors that were not involved in performing the stainings.

Behavioural & social sciences study design

All studies must disclose on these points even when the disclosure is negative.

Study description	*Briefly describe the study type including whether data are quantitative, qualitative, or mixed-methods (e.g. qualitative cross-sectional, quantitative experimental, mixed-methods case study).*
Research sample	*State the research sample (e.g. Harvard university undergraduates, villagers in rural India) and provide relevant demographic information (e.g. age, sex) and indicate whether the sample is representative. Provide a rationale for the study sample chosen. For studies involving existing datasets, please describe the dataset and source.*
Sampling strategy	*Describe the sampling procedure (e.g. random, snowball, stratified, convenience). Describe the statistical methods that were used to predetermine sample size OR if no sample-size calculation was performed, describe how sample sizes were chosen and provide a rationale for why these sample sizes are sufficient. For qualitative data, please indicate whether data saturation was considered, and what criteria were used to decide that no further sampling was needed.*
Data collection	*Provide details about the data collection procedure, including the instruments or devices used to record the data (e.g. pen and paper, computer, eye tracker, video or audio equipment) whether anyone was present besides the participant(s) and the researcher, and whether the researcher was blind to experimental condition and/or the study hypothesis during data collection.*
Timing	*Indicate the start and stop dates of data collection. If there is a gap between collection periods, state the dates for each sample cohort.*

Data exclusions	*If no data were excluded from the analyses, state so OR if data were excluded, provide the exact number of exclusions and the rationale behind them, indicating whether exclusion criteria were pre-established.*
Non-participation	*State how many participants dropped out/declined participation and the reason(s) given OR provide response rate OR state that no participants dropped out/declined participation.*
Randomization	*If participants were not allocated into experimental groups, state so OR describe how participants were allocated to groups, and if allocation was not random, describe how covariates were controlled.*

Ecological, evolutionary & environmental sciences study design

All studies must disclose on these points even when the disclosure is negative.

Study description	*Briefly describe the study. For quantitative data include treatment factors and interactions, design structure (e.g. factorial, nested, hierarchical), nature and number of experimental units and replicates.*
Research sample	*Describe the research sample (e.g. a group of tagged Passer domesticus, all Stenocereus thurberi within Organ Pipe Cactus National Monument), and provide a rationale for the sample choice. When relevant, describe the organism taxa, source, sex, age range and any manipulations. State what population the sample is meant to represent when applicable. For studies involving existing datasets, describe the data and its source.*
Sampling strategy	*Note the sampling procedure. Describe the statistical methods that were used to predetermine sample size OR if no sample-size calculation was performed, describe how sample sizes were chosen and provide a rationale for why these sample sizes are sufficient.*
Data collection	*Describe the data collection procedure, including who recorded the data and how.*
Timing and spatial scale	*Indicate the start and stop dates of data collection, noting the frequency and periodicity of sampling and providing a rationale for these choices. If there is a gap between collection periods, state the dates for each sample cohort. Specify the spatial scale from which the data are taken*
Data exclusions	*If no data were excluded from the analyses, state so OR if data were excluded, describe the exclusions and the rationale behind them, indicating whether exclusion criteria were pre-established.*
Reproducibility	*Describe the measures taken to verify the reproducibility of experimental findings. For each experiment, note whether any attempts to repeat the experiment failed OR state that all attempts to repeat the experiment were successful.*
Randomization	*Describe how samples/organisms/participants were allocated into groups. If allocation was not random, describe how covariates were controlled. If this is not relevant to your study, explain why.*
Blinding	*Describe the extent of blinding used during data acquisition and analysis. If blinding was not possible, describe why OR explain why blinding was not relevant to your study.*

Did the study involve field work? ☐ Yes ☐ No

Field work, collection and transport

Field conditions	*Describe the study conditions for field work, providing relevant parameters (e.g. temperature, rainfall).*
Location	*State the location of the sampling or experiment, providing relevant parameters (e.g. latitude and longitude, elevation, water depth).*
Access and import/export	*Describe the efforts you have made to access habitats and to collect and import/export your samples in a responsible manner and in compliance with local, national and international laws, noting any permits that were obtained (give the name of the issuing authority, the date of issue, and any identifying information).*
Disturbance	*Describe any disturbance caused by the study and how it was minimized.*

Reporting for specific materials, systems and methods

Materials & experimental systems

n/a	Involved in the study
☐	☒ Unique biological materials
☐	☒ Antibodies
☐	☒ Eukaryotic cell lines
☒	☐ Palaeontology
☐	☒ Animals and other organisms
☒	☐ Human research participants

Methods

n/a	Involved in the study
☒	☐ ChIP-seq
☐	☒ Flow cytometry
☒	☐ MRI-based neuroimaging

Unique biological materials

Policy information about availability of materials

Obtaining unique materials: Human osteosarcoma U2OS cells expressing GFP-LC3, CALR-RFP or HMGB1-GFP were generated in the lab and fully described in previous publication (DOI: 10.1126/scitranslmed.3003807); The Published Kinase Inhibitor Set (PKIS) and the complementary set, PKIS2 were kindly shared by SGC-UNC (Structural Genomics Consortium-UNC Eshelman School of Pharmacy at the University of North Carolina, Raleigh-Durham, NC, USA).

Antibodies

Antibodies used: anti-CALR antibody (ab2907), anti-p-EIF2α (ab32157), anti-EIF2α (ab5369), anti-ATF4 (ab31390), anti-XBP1 (ab37152), anti-β-Actin (ab20272), Anti-CD3 antibody (ab5690) and Anti-CD11c antibody (ab11029) were purchased from Abcam; anti-Hexokinase II (2867) were purchased from Cell Signaling Technology; Alexa Fluor® 488-conjugated anti-mouse CD86 antibody (clone GL-1), Alexa Fluor® 488 or Alexa Fluor® 594 -conjugated anti-mouse CD8a antibody (clone 53-6.7), Pacific Blue anti-mouse CD8a (Clone 53-6.7), APC anti-mouse CD3ε (Clone 145-2C1), Alexa Fluor® 488-conjugated anti-mouse Foxp3 antibody (clone MF-14) and PE-Cy7-conjugated anti-mouse CD152 / CTLA-4 antibody (clone UC10-4B9) were purchased from BioLegend. BV421-conjugated anti-mouse CD278/ICOS (clone 7E.17G9), FITC or PE-conjugated anti-mouse CD8a (clone 53-6.7), FITC anti-mouse CD4 (RM4-4), PE and APC anti-mouse IFN? (XMG1.2), PE Rat anti-mouse IL4 (1B11), BV605 anti-mouse IL17A (TC11-18H10), PE-Cy7-conjugated anti-mouse CD11c (clone HL3) were purchased from BD Bioscience. PerCP-Cy5.5 or pacific blue-conjugated anti-mouse CD4 (clone RM4-5), PE-Cy7-conjugated anti-mouse CD25 (clone PC61 5.3), Foxp3 efluor450 (Clone FJS-16s), APC or FITC-conjugated anti-mouse CD3g,d,e (clone 17A2), APC-eFluor780, APC-Cy7-conjugated anti-mouse CD279 / PD-1 (clone J43), PE-conjugated anti-mouse IL-17A (clone eBio17B7), Percp_Cy5.5-conjugated anti-mouse CD3e (clone 145-2C11), FITC-conjugated anti-mouse LAG-3 (clone eBioC9B7W), PE-conjugated anti-mouse PD-1 (clone J43), APC-conjugated anti-mouse TIM-3 (clone 8B.2C12), APC-conjugated anti-mouse TIM-3 (clone 8B.2C12), pacific blue-conjugated anti-mouse CD11b (clone M1/70.15), Alexa Fluor® 488-conjugated anti-mouse Ly-6C (clone HK1.4), and PE-conjugated anti-mouse Ly-6G (clone 1A8-Ly6g), anti-mouse CD274 (PD-L1, clone MIH1), anti-mouse MHC Class I (H-2Kb, clone AF6-88.5.5.3), anti-mouse MHC Class II (I-A/I-E, clone M5/114.15.2) were purchased from eBioscience. In vivo neutralizing antibodies to PD-1 (CD279, clone 29F.1A12), CTLA-4 (CD152, clone 9D9), IFNAR-1(clone MAR1-5A3), IL-12 p40 (clone C17.8), IFNγ (clone R4-6A2), CD4 (clone GK1.5), CD8 (clone 2.43), CD11b (clone M1/70) as well as corresponding isotype antibodies (Clone LTF-2, 2A3, MOPC-21, HRPN,) were purchased from BioXcell.

Validation: Validation of the commercially available antibodies can be found in their product page following the clone numbers provided.

Eukaryotic cell lines

Policy information about cell lines

Cell line source(s): Unless specified, all wild-type cancer cell lines were purchased from the American Type Culture Collection (ATCC, Rockefeller, MD, USA)

Authentication: Authentication of commercially available cell lines were provided by ATCC, gene edited cell lines have been validated by western blotting. Biosensor cells were derived from authenticated wild type cells and were maintained carefully. All cell morphology have been validated identical to published images.

Mycoplasma contamination: Mycoplasma contamination has been regularly tested during all studies and has been validated negative before launching experiments.

Commonly misidentified lines (See ICLAC register): No commonly misidentified cell lines were used.

Palaeontology

Specimen provenance: *Provide provenance information for specimens and describe permits that were obtained for the work (including the name of the issuing authority, the date of issue, and any identifying information).*

Specimen deposition: *Indicate where the specimens have been deposited to permit free access by other researchers.*

Dating methods: *If new dates are provided, describe how they were obtained (e.g. collection, storage, sample pretreatment and measurement),*

Dating methods

where they were obtained (i.e. lab name), the calibration program and the protocol for quality assurance OR state that no new dates are provided.

☐ Tick this box to confirm that the raw and calibrated dates are available in the paper or in Supplementary Information.

Animals and other organisms

Policy information about studies involving animals; ARRIVE guidelines recommended for reporting animal research

Laboratory animals

Six- to eight-week-old female wild-type C57BL/6 mice were obtained from ENVIGO France (Gannat, France). Six- to seven-week-old female athymic nude (nu/nu) mice were obtained from Harlan France (Gannat, France). All animal experiments using C57BL/6 mice or nude mice were performed in compliance with the EU Directive 63/2010 and specific ethic protocol (Protocols 2016_082 that was approved by the Ethical Committee of the Gustave Roussy Campus Cancer, CEEA IRCIV/IGR no. 26, registered at the French Ministry of Research). Naive FVB mice (female, aged between 6-7 weeks, weighing 20-22 g) were purchased from Charles River, Beijing, China. Protocol using FVB mice was approved by the Ethics Committee of Suzhou Institute of Systems Medicine, Chinese Academy of Medical Sciences (No: 2017AWEC011). KP mice (129 background) were bred in the Pittet Lab at the Massachusetts General Hospital, Harvard Medical School. Related animal experiments were approved by the Massachusetts General Hospital Subcommittee on Research Animal Care.

Wild animals

No wild animals have been involved in this study.

Field-collected samples

No samples collected from the field have been involved in this study.

Human research participants

Policy information about studies involving human research participants

Population characteristics

Describe the covariate-relevant population characteristics of the human research participants (e.g. age, gender, genotypic information, past and current diagnosis and treatment categories). If you filled out the behavioural & social sciences study design questions and have nothing to add here, write "See above."

Recruitment

Describe how participants were recruited. Outline any potential self-selection bias or other biases that may be present and how these are likely to impact results.

ChIP-seq

Data deposition

☐ Confirm that both raw and final processed data have been deposited in a public database such as GEO.

☐ Confirm that you have deposited or provided access to graph files (e.g. BED files) for the called peaks.

Data access links
May remain private before publication.

For "Initial submission" or "Revised version" documents, provide reviewer access links. For your "Final submission" document, provide a link to the deposited data.

Files in database submission

Provide a list of all files available in the database submission.

Genome browser session
(e.g. UCSC)

Provide a link to an anonymized genome browser session for "Initial submission" and "Revised version" documents only, to enable peer review. Write "no longer applicable" for "Final submission" documents.

Methodology

Replicates

Describe the experimental replicates, specifying number, type and replicate agreement.

Sequencing depth

Describe the sequencing depth for each experiment, providing the total number of reads, uniquely mapped reads, length of reads and whether they were paired- or single-end.

Antibodies

Describe the antibodies used for the ChIP-seq experiments; as applicable, provide supplier name, catalog number, clone name, and lot number.

Peak calling parameters

Specify the command line program and parameters used for read mapping and peak calling, including the ChIP, control and index files used.

Data quality

Describe the methods used to ensure data quality in full detail, including how many peaks are at FDR 5% and above 5-fold enrichment.

Software

Describe the software used to collect and analyze the ChIP-seq data. For custom code that has been deposited into a community repository, provide accession details.

Flow Cytometry

Plots

Confirm that:

☒ The axis labels state the marker and fluorochrome used (e.g. CD4-FITC).

☒ The axis scales are clearly visible. Include numbers along axes only for bottom left plot of group (a 'group' is an analysis of identical markers).

☒ All plots are contour plots with outliers or pseudocolor plots.

☒ A numerical value for number of cells or percentage (with statistics) is provided.

Methodology

Sample preparation	Human cancer cells were collected by trypsin. tumors were excised and immediately collected in gentleMACS C Tubes (Miltenyi Biotech; Bergisch Gladbach, Germany) containing 1 mL RPMI-1640 medium. The samples were kept on ice until dissociation using the gentleMACS Dissociator with a Miltenyi mouse tumor dissociation kit (Miltenyi Biotech) according to the manufacturer's protocol. The dissociated bulk tumor cell suspension was resuspended in RPMI-1640, sequentially passed through 70 µm and 30 µm nylon cell strainers (Miltenyi Biotec) and washed twice with cold PBS.<br>Peripheral blood mononuclear cells (PBMCs) were isolated by Ficoll-Paque PLUS (GE Healthcare) density gradient centrifugation.
Instrument	BD LSRFortessa Flow Cytometer; MACSQuant® Analyzer 10
Software	BD FACSDiva, MACSQuant®
Cell population abundance	No sorting involved in this study.
Gating strategy	Initial cell population gating was placed on FSC / SSC to exclude nonlymphocytes and LIVE/DEAD® Fixable Yellow Dead Cell dye (Invitrogen) was used to discriminate viable cells from damaged/dead cells for downstream analysis. Detailed gating strategy can be found in supplementary figures 10, 11, and 16

☒ Tick this box to confirm that a figure exemplifying the gating strategy is provided in the Supplementary Information.

Magnetic resonance imaging

Experimental design

Design type	*Indicate task or resting state; event-related or block design.*
Design specifications	*Specify the number of blocks, trials or experimental units per session and/or subject, and specify the length of each trial or block (if trials are blocked) and interval between trials.*
Behavioral performance measures	*State number and/or type of variables recorded (e.g. correct button press, response time) and what statistics were used to establish that the subjects were performing the task as expected (e.g. mean, range, and/or standard deviation across subjects).*

Acquisition

Imaging type(s)	*Specify: functional, structural, diffusion, perfusion.*
Field strength	*Specify in Tesla*
Sequence & imaging parameters	*Specify the pulse sequence type (gradient echo, spin echo, etc.), imaging type (EPI, spiral, etc.), field of view, matrix size, slice thickness, orientation and TE/TR/flip angle.*
Area of acquisition	*State whether a whole brain scan was used OR define the area of acquisition, describing how the region was determined.*

Diffusion MRI ☐ Used ☐ Not used

Preprocessing

Preprocessing software	*Provide detail on software version and revision number and on specific parameters (model/functions, brain extraction, segmentation, smoothing kernel size, etc.).*
Normalization	*If data were normalized/standardized, describe the approach(es): specify linear or non-linear and define image types used for transformation OR indicate that data were not normalized and explain rationale for lack of normalization.*
Normalization template	*Describe the template used for normalization/transformation, specifying subject space or group standardized space (e.g. original Talairach, MNI305, ICBM152) OR indicate that the data were not normalized.*

Noise and artifact removal

Describe your procedure(s) for artifact and structured noise removal, specifying motion parameters, tissue signals and physiological signals (heart rate, respiration).

Volume censoring

Define your software and/or method and criteria for volume censoring, and state the extent of such censoring.

Statistical modeling & inference

Model type and settings

Specify type (mass univariate, multivariate, RSA, predictive, etc.) and describe essential details of the model at the first and second levels (e.g. fixed, random or mixed effects; drift or auto-correlation).

Effect(s) tested

Define precise effect in terms of the task or stimulus conditions instead of psychological concepts and indicate whether ANOVA or factorial designs were used.

Specify type of analysis: ☐ Whole brain ☐ ROI-based ☐ Both

Statistic type for inference
(See Eklund et al. 2016)

Specify voxel-wise or cluster-wise and report all relevant parameters for cluster-wise methods.

Correction

Describe the type of correction and how it is obtained for multiple comparisons (e.g. FWE, FDR, permutation or Monte Carlo).

Models & analysis

n/a	Involved in the study
☐	☐ Functional and/or effective connectivity
☐	☐ Graph analysis
☐	☐ Multivariate modeling or predictive analysis

Functional and/or effective connectivity

Report the measures of dependence used and the model details (e.g. Pearson correlation, partial correlation, mutual information).

Graph analysis

Report the dependent variable and connectivity measure, specifying weighted graph or binarized graph, subject- or group-level, and the global and/or node summaries used (e.g. clustering coefficient, efficiency, etc.).

Multivariate modeling and predictive analysis

Specify independent variables, features extraction and dimension reduction, model, training and evaluation metrics.
