# Supplementary material for: Crizotinib-induced immunogenic cell death in non-small cell lung cancer
Source: Nat Commun. 2019 Apr 2;10:1486. doi: 10.1038/s41467-019-09415-3 (PMC6445096; doi:10.1038/s41467-019-09415-3)
Supplement: Supplementary file 7 — Description of Additional Supplementary Files [file 41467_2019_9415_MOESM7_ESM.docx]

**Title: Supplementary Data1**.
**Description:** Mean Z-scores of ICD parameters obtained screening the Public Chemogenomic Set for Protein Kinases.

**Title: Supplementary Data2**.
**Description:** Mean Z-scores of ICD parameters obtained screening a collection of tyrosine kinase inhibitors.

**Title: Supplementary Data3**.
**Description:** Statistical evaluation extracted from RNA-Seq analysis. Sheet1, fold change and adjusted *P* values of genes regulated in response to treatment with (R)-crizotinib + cisplatin; Sheet2, Gene symbols clustered according to Gene Ontology terms.
